# Aqueously Upcycled Lignin with Emergent Tribonegativity for Skin‐Integrated Triboelectronics

**DOI:** 10.1002/adma.202518412

**Published:** 2025-12-09

**Authors:** Robert Ccorahua‐Santo, Mi Li, Yi Zheng, Wenzhuo Wu

**Affiliations:** ^1^ Edwardson School of Industrial Engineering Purdue University West Lafayette IN 47907 USA; ^2^ Flex Laboratory Purdue University West Lafayette IN 47907 USA; ^3^ Center for Renewable Carbon School of Natural Resources University of Tennessee Knoxville, 2506 Jacob Drive Knoxville TN 37996‐4570 USA; ^4^ Department of Grain Science and Industry Kansas State University 101C BIVAP, 1980 Kimball Avenue Manhattan KS 66506 USA; ^5^ Women's Global Health Institute Purdue University West Lafayette IN 47907 USA; ^6^ Purdue Institute for Integrative Neuroscience Purdue University West Lafayette IN 47907 USA; ^7^ Purdue Institute of Inflammation Immunology, and Infectious Disease Purdue University West Lafayette IN 47907 USA; ^8^ Purdue Institute for Drug Discovery Purdue University West Lafayette IN 47907 USA; ^9^ Institute for Physical AI Purdue University West Lafayette IN 47907 USA

**Keywords:** skin‐integrated sensors, triboelectric nanogenerators, triboelectric sensors, tribonegativity, upcycled lignin

## Abstract

Valorizing lignin, a vast industrial byproduct and abundant biomass, is critical for a circular bioeconomy. However, the potential of lignin as a feedstock for functional polymers remains unrealized owing to poor aqueous solubility. Herein, a scalable aqueous process is reported that transforms lignin into printable electronic ink. The benign urea‐based formulation increases lignin dispersibility by two orders of magnitude to 100 mg mL^−1^, while preserving its molecular integrity by retaining 97.6% of its fragile β‐O‐4′ ether linkages. This process enables the thermodynamically driven self‐assembly of lignin polymers during printing to create a functional, nanotextured surface with emergent tribonegativity, without the use of harsh solvents or lithography. As a proof of concept, skin‐integrated triboelectric sensors fabricated from this ink generate high‐fidelity signals sufficient for objectively classifying human mental workload, with a performance comparable to gold‐standard electrocardiography. This study establishes a generalizable strategy for creating high‐performance, sustainable electronics from waste biomass.

## Graphical abstract

Aqueously upcycled lignin is transformed into a printable ink that self‐assembles into a nanotextured surface during printing with the highest reported biopolymer‐based tribonegativity. The resulting skin‐integrated triboelectric sensor enables high‐fidelity cardiovascular monitoring and objective mental workload classification, demonstrating a scalable route to high‐performance, sustainable electronics from otherwise wasted biomass.

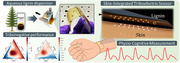

## 1 Introduction

Valorizing lignin, the second most abundant biopolymer on earth and a vast underutilized waste stream from the paper industry, represents a cornerstone of the circular bioeconomy.^[^ ^1^ ^]^ Annually, over 98% of the tens of millions of tons of lignin produced is incinerated as low‐grade fuel, forfeiting its immense potential as a sustainable chemical feedstock.^[^ ^2^ ^]^ Although the aromatic structure of lignin is an ideal platform for functional polymers,^[^ ^3^ ^]^ this potential has not been realized because of a critical processing barrier: its poor solubility in benign solvents necessitates harsh processing methods that irreversibly degrade its molecular integrity and, consequently, its electronic and mechanical properties.^[^ ^4^, ^5^ ^]^ Overcoming this processing challenge is crucial for resolving the fundamental trade‐off between performance and sustainability that currently plagues applications such as wearable electronics.^[^ ^6^ ^]^ For example, high‐performance skin‐integrated triboelectronics require materials with strong tribonegativity to generate robust signals against tribopositive human skin.^[^ ^7^, ^8^ ^]^ This function is currently fulfilled by unsustainable synthetic fluoropolymers, such as polytetrafluoroethylene (PTFE), whereas most sustainable biopolymers, including cellulose^[^ ^9^, ^10^ ^]^ and chitosan,^[^ ^11^ ^]^ are typically tribopositive or weakly tribonegative. The aromatic‐rich structure of lignin is inherently suited to enhance its tribonegativity. Yet, its potential has remained inaccessible and locked away by the same processing barrier that prevents its general valorization.^[^ ^12^ ^]^

Here, we overcome this processing barrier with a scalable aqueous process, guided by Hansen solubility parameter analysis,^[^ ^13^ ^]^ to direct the thermodynamically driven self‐assembly of lignin films. This process‐as‐design strategy creates aromatic‐rich charge‐enhancing domains on the nanotextured surface with emergent tribonegativity that rivals other biopolymers and is comparable to that of synthetic polymers, a feat achieved while preserving the molecular integrity of lignin. We fabricated a proof‐of‐concept skin‐integrated triboelectric sensor to demonstrate the effectiveness of this strategy. The resulting sensor captures high‐fidelity cardiovascular waveforms from the wrist and objectively classifies the human mental workload with performance comparable to gold‐standard electrocardiography (ECG) measurements. This work establishes a comprehensive material‐to‐system pathway for biomass valorization, presents a generalizable strategy for creating high‐performance functional materials from waste biomass, and opens a new route for sustainable and circular manufacturing in fields such as wearable electronics.

## 2 Results

### 2.1 Rational Design Unlocks High‐Concentration Aqueous Lignin Processing

**Figure 1:**
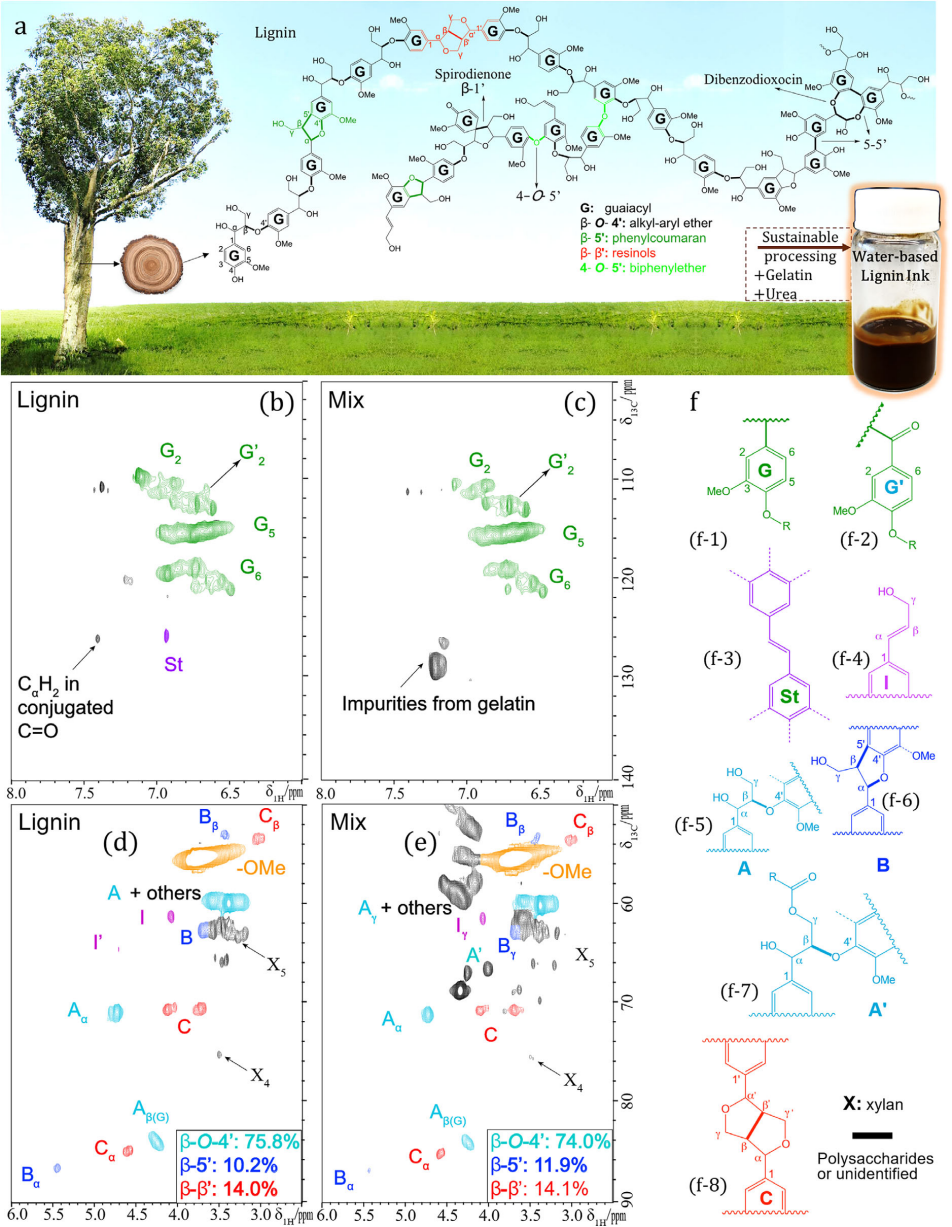
Benign aqueous processing of kraft lignin preserves its molecular integrity. a) Schematic of the lignin molecular structure and dispersed lignin ink. b–e) Comparative 2D‐HSQC‐NMR spectra of pure lignin and the lignin mixture in the aromatic b,c) and aliphatic d,e) regions. The spectra provided definitive evidence of structural preservation, confirming the retention of both the key aromatic guaiacyl (G, G’) units and, critically, 97.6% of the fragile β‐O‐4′ ether linkages A). f) The primary lignin substructures identified and quantified in the NMR spectra, including the molecular structures of lignin monolignol units: guaiacyl (G) and oxidized guaiacyl (G’) (1,2), and the principal aliphatic groups of lignin (specific C‐H pairs of St: Stilbene (3), I: cinnamyl alcohol (4), A: β‐O‐4′ linkage (5), B: β‐5′ linkage (6), A’: γ‐acylated β‐O‐4′ linkage (7), and C: β‐β’ linkage (8).

The cornerstone of our strategy is a printable aqueous ink that overcomes the longstanding processing barrier of softwood pine kraft lignin (**Figure** **1**a). This biopolymer is mainly composed of non‐polar aromatic guaiacyl (G) units linked via β‐O‐4′ ether bonds and polar groups, such as hydroxyls and carboxyls.^[^ ^14^ ^]^ Although the aromaticity of lignin makes it an ideal tribonegative material,^[^ ^15^ ^]^ its practical application has been prevented by its poor aqueous dispersibility (≈1 mg mL^−1^), which is incompatible with scalable coating processes.^[^ ^16^ ^]^ To overcome this, we developed a formulation that increased lignin dispersibility by two orders of magnitude to 100 mg mL^−1^, a 100‐fold increase over distilled water^[^ ^16^ ^]^ and a 10‐fold increase over alkaline solutions,^[^ ^17^ ^]^ without lignin depolymerization. The rational selection of urea and gelatin as key components was guided by Hansen Solubility Parameter (HSP) analysis (Figures S1,S2, Supporting Information), a predictive framework that assesses molecular compatibility based on dispersion (δ_D_), polar (δ_P_), and hydrogen bonding (δ_H_) forces.^[^ ^13^ ^]^ The analysis revealed a close match between the HSP of lignin (δ_D_ 21.9, δ_P_ 14.1, δ_H_ 16.9) and urea (δ_D_ 22.9, δ_P_ 14.9, δ_H_ 21.3), explaining the critical dual functionality of urea. In the aqueous phase, urea acts as a hydrotrope, disrupting the intermolecular self‐association of lignin and mediating its interaction with water.^[^ ^18^ ^]^ In the solid state, urea functions as a molecular plasticizer, preventing large‐scale phase segregation between lignin and gelatin binder.^[^ ^19^ ^]^ Gelatin was selected as a binder to ensure film integrity via intermolecular hydrogen bonding and aromatic non‐polar interactions.^[^ ^20^ ^]^ This rational, theory‐driven formulation yields printable, high‐concentration aqueous ink without resorting to harsh solvents or lignin depolymerization.^[^ ^16^, ^17^ ^]^

### 2.2 Benign Aqueous Processing Preserves Lignin's Molecular Integrity

A key advantage of our benign aqueous process is the preservation of the native molecular structure of lignin, which is a critical prerequisite for achieving both a high triboelectric performance and biocompatibility.^[^ ^12^, ^21^ ^]^ Typical dispersion methods can unintentionally depolymerize lignin to molecular weights below 500 Da,^[^ ^5^ ^]^ a threshold for potential skin penetration.^[^ ^21^ ^]^ To provide evidence of this preservation, we employed 2D heteronuclear single quantum coherence (2D‐HSQC) NMR spectroscopy, which characterizes the structure of complex molecules, such as the original lignin and lignin in the mixture, and identified different structural units and linkages within the lignin polymer (Figure 1b–e; Figures S3,S4, Supporting Information).^[^ ^22^ ^]^ Figure 1b–e shows the 2D‐HSQC‐NMR spectra of lignin and lignin mixture in the aliphatic (δ_C_/δ_H_ 50–90/2.5–6.0 ppm) and aromatic (δ_C_/δ_H_ 95–135/6.0–8.0 ppm) regions, respectively, providing information on the subunits and linkages of the lignin molecule^[^ ^23^ ^]^ (Figure S3, Supporting Information). The presence of lignin, urea, and gelatin was verified using full ^1^H‐NMR and 2D HSQC‐NMR spectroscopy, as shown in Figures S3,S4 (Supporting Information). Figure S3 (Supporting Information) shows the characteristic spectral patterns of the three components, and Figure S4 (Supporting Information) shows the full 2D‐HSQC‐NMR spectra of the original lignin and the mixture, along with detailed descriptions of the aliphatic and aromatic regions. The analysis confirmed the full preservation of the key aromatic guaiacyl units (specific C─H cross‐peaks labeled G_2_, G’_2_, G_5_, and G_6_), which are essential for tribonegativity^[^ ^24^ ^]^ (Figure 1b,c). The spectra show that the primary aliphatic linkages of lignin are present in both the original and mixed samples (cross‐peaks A: β‐O‐4′, A’: γ‐acylated β‐O‐4′, B: β‐5′, and C: β‐β’) (Figure 1d,e). The data revealed a retention of 97.6% of fragile β‐O‐4′ ether linkages (74.0% abundance versus 75.8% in the original lignin), which are notoriously susceptible to cleavage during conventional harsh chemical treatments.^[^ ^25^ ^]^ The more stable C‐C bonds (β‐β’ and β‐5′) were also well‐preserved (Figure 1f). The remarkable preservation of the polymer backbone is vital. It maintains a high molecular weight (>1.1 kDa), safely above the 500 Da skin penetration threshold,^[^ ^5^ ^]^ and ensures the structural stability required for robust and reliable device performance.^[^ ^4^ ^]^

### 2.3 Process‐Driven Self‐Assembly Creates a Functional Triboelectric Surface

**Figure 2:**
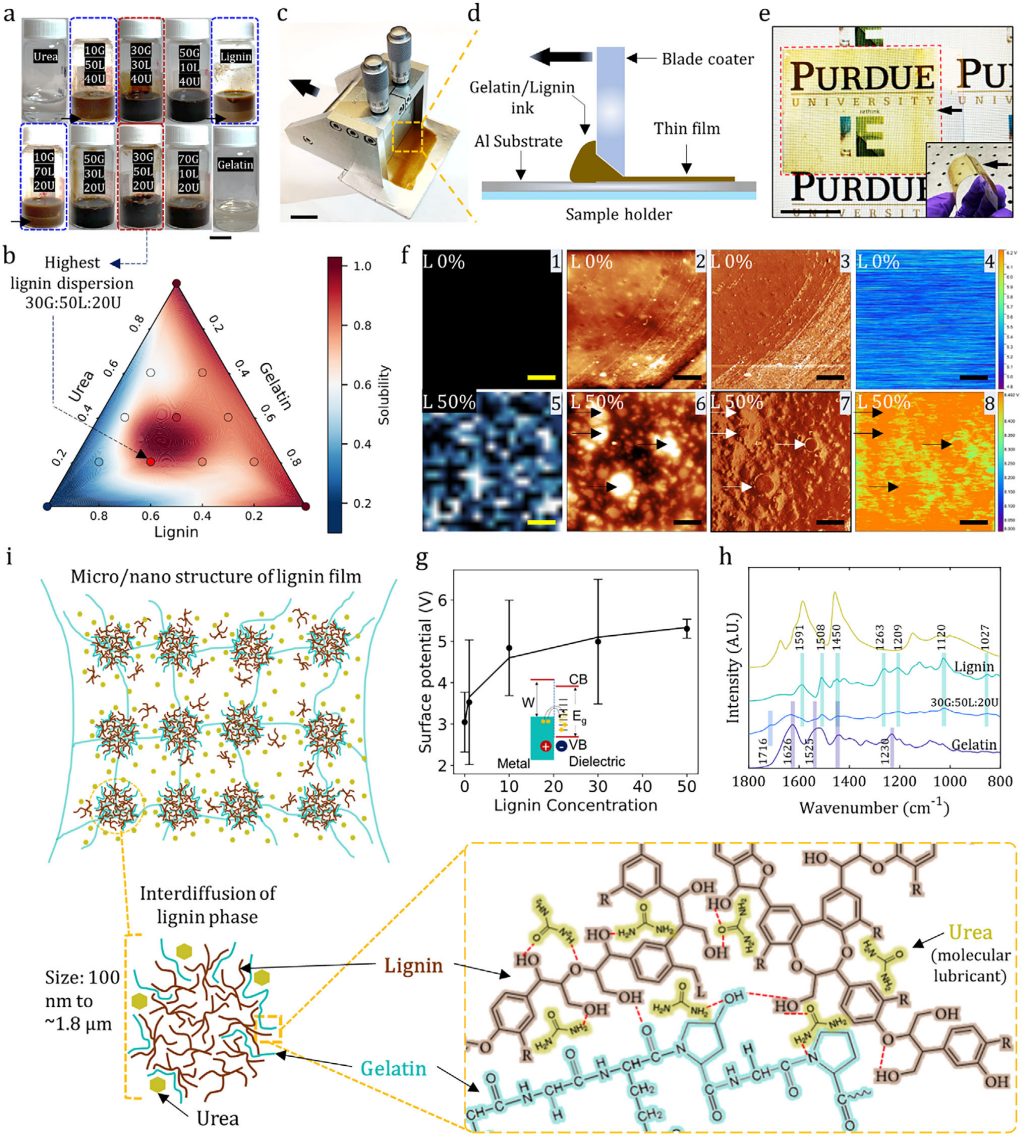
Process‐driven self‐assembly creates a functional triboelectric surface. a–e) Ink optimization and film fabrication. A ternary phase diagram b) identifies the optimal ink composition (≈30G:50L:20U) for high dispersibility a), which was processed via a scalable blade‐coating method c,d) into flexible thin films e). f,g) Direct correlation between process‐derived structure and emergent function. Correlated AFM and KPFM f) provide direct evidence that self‐assembled lignin‐rich nanodomains are regions with high negative surface potentials. This is quantified in g), showing that increasing the lignin concentration creates a strongly negative surface, which is the physical origin of the emergent tribonegativity. h) Spectroscopic evidence of the stabilization mechanism. ATR‐FTIR spectra revealed the formation of new intermolecular hydrogen bonds (arrow, 1716 cm^−1^) that kinetically stabilized the functional nanostructure. i) A unified mechanistic model summarizing the pathway from a rationally designed aqueous ink to a functional, stabilized surface via thermodynamically driven self‐assembly.

We first optimized the ink composition by evaluating the gravitational dispersibility across various weight ratios of gelatin (G), lignin (L), and urea (U) (**Figure** **2**a). A ternary phase diagram revealed that the dispersibility increased with the introduction of gelatin and urea, with the highest degree of dispersion (0.8) achieved at a 30G: 50 L:20U ratio (Figure 2b, Figure S5, Supporting Information). This optimized ink was then blade‐coated to form homogeneous ultrathin (≈1 µm) films (Figure 2c–e), initiating a thermodynamically driven self‐assembly process. The printed lignin film exhibited transparency and good homogeneity on the PET substrate, indicating that no macroscale segregation occurred during blade coating (Figure 2e). During solvent evaporation, the incompatibility between the nonpolar lignin and polar gelatin drives phase separation to minimize the Gibbs free energy of the system, dictating the final surface morphology.^[^ ^26^ ^]^ Correlated atomic force microscopy (AFM) and fluorescence microscopy provided direct evidence of this self‐assembly, revealing that lignin segregates into protruding, lignin‐rich nanodomains on the film surface (Figure 2f; Figures S6–S8, Supporting Information).^[^ ^27^ ^]^ These images show the correlation between the intrinsic fluorescence of lignin and nanodomain distribution on the surface of the films (Figure 2f1,f5). These domains, which are absent in pure gelatin films, had lateral sizes of 100 nm to ≈1.8 µm and heights of 30–140 nm (Figure S9, Supporting Information). In contrast, the pure gelatin films exhibited flat surfaces without protrusions or aggregations (Figure 2f2). In addition, nanodomains appeared only at high lignin concentrations (Figure 2f6). As described above, because the nanodomains and fluorescence correlate in the films, it is suggested that lignin is primarily present in the nanodomains rather than in the interstitial space between them. This emergent nanotexture on the surface of films coated with the optimized ink, formed via an enthalpically driven dewetting process,^[^ ^4^ ^]^ is the direct origin of the enhanced tribological properties of the material and is achieved without resorting to complex lithography.

To investigate the direct link between process‐derived morphology and its function, we employed Kelvin probe force microscopy (KPFM) to map the surface potential (SP).^[^ ^28^ ^]^ The resulting surface potential map revealed a definitive spatial correlation between the lignin‐rich nanodomains identified by AFM and regions of high negative surface potential (Figure 2f). This correlation is quantified by the average surface potential, which becomes progressively more negative with increasing lignin concentration, shifting from 2.32 eV for pure gelatin to 5.5 eV for the optimized 50% lignin film (Figure 2g; Figures S9–S11, Supporting Information). This provides a clear physical mechanism for the emergent tribonegativity. To explain the triboelectrification phenomenon in lignin using KPFM analysis, we employed a metal‐dielectric contact model (Figure 2g inset).^[^ ^8^ ^]^ When gold from the cantilever tip in KPFM and lignin come into contact with each other, surface charge transfer occurs at the contact area owing to contact electrification, resulting in lignin gaining electrons on its surface and gold losing electrons from its surface. The profile of the scanned region, as provided by KPFM, shows the SP distribution across the area, indicating that the lignin surface has acquired negative electrostatic charges and hence has a high electron affinity^[^ ^28^ ^]^ (Figure S10, Supporting Information). The aromatic groups in lignin molecules have high electron affinity,^[^ ^29^ ^]^ unlike polar side groups (such as hydroxyl, carboxyl, and ether), which have lower electron affinities.^[^ ^30^ ^]^ This high electron affinity has been observed for synthetic polymers with high aromatic content, such as polyimide and polystyrene.^[^ ^31^ ^]^ The self‐assembly process concentrates the high‐electron‐affinity aromatic groups of lignin onto protruding surface domains. These domains function as charge‐accumulating “hotspots” that are the origin of the enhanced contact electrification against the tribopositive human skin (Figure S10, Supporting Information).

The mechanism stabilizing this functional nanostructure, which dictates the triboelectric properties of the formed lignin films, was elucidated using attenuated total reflectance Fourier transform infrared (ATR‐FTIR) spectroscopy (Figure 2h).^[^ ^32^ ^]^ The spectra confirm the presence of the characteristic aromatic groups of lignin that are essential for its electron‐withdrawing capability (e.g., aromatic skeletal vibrations at 1591 and 1508 cm^−1^)^[^ ^33^ ^]^ (Figure 2h, green shaded regions). Other functional polar groups, such as hydroxyl and carboxyl groups, were overshadowed by signals from the water molecules and aromatic groups.^[^ ^34^ ^]^ The band at 1450 cm^−1^ corresponds to C─H in‐plane deformation with aromatic ring stretching. The bands at 1263 and 1209 cm^−1^ correspond to the C─O and C─C stretching of the guaiacyl unit, respectively. The 1120 and 1027 cm^−1^ bands belong to aromatic C─H in‐plane deformation (guaiacyl ring). Moreover, the vibration bands of pure gelatin (purple curve) and gelatin in the mixture were identified. The 1626 and 1525 cm^−1^ vibration bands correspond to amide I (C═O stretching vibration) and amide II (N─H bending), respectively.^[^ ^33^ ^]^ The intensity of the amide bands of gelatin was reduced, possibly because of the hydrogen bond interactions between lignin and gelatin, as described below. Critically, these results also reveal key stabilizing interactions. A new C═O stretching band appeared at 1716 cm^−1^, an absorption feature present exclusively in the 30G: 50 L:20U film. Its absence from the spectra of the individual components provides definitive evidence for the formation of new intermolecular hydrogen bonds between the carbonyl groups of gelatin (whose amide I and II bands are correspondingly reduced) and the hydroxyl groups of lignin.^[^ ^32^, ^35^ ^]^ These hydrogen bonds are essential for kinetically stabilizing the phase‐separated nanostructure observed using AFM (Figure 2f), thereby preventing large‐scale aggregation^[^ ^36^ ^]^ and ensuring the mechanical integrity required for a robust device. In addition, unlike the tendency of lignin molecules to aggregate in pure water,^[^ ^37^ ^]^ the presence of the 1716 cm^−1^ band and strong aromatic bands between 1450 and 1600 cm^−1^ suggests that lignin can be dispersed with gelatin and urea in a solid lignin film.

These combined microscopic and spectroscopic findings provide a complete mechanistic picture of the formation of a hierarchical triboelectric surface via process‐driven self‐assembly (Figure 2i). The process begins with a rationally designed aqueous ink, where urea acts as a hydrotrope to create a stable, high‐concentration lignin dispersion. During coating and drying, thermodynamically driven phase separation induces self‐assembly of protruding aromatic‐rich nanodomains. These domains create surface potential hotspots that are the origin of the material's strong tribonegativity. Finally, in the solid state, synergistic stabilization occurs, where urea functions as a molecular plasticizer, mediating interdiffusion. At the same time, intermolecular hydrogen bonds and dipole‐dipole interactions between lignin and gelatin kinetically stabilize the nanostructure. This dual mechanism prevents the large‐scale segregation common in lignin polymer blends^[^ ^38^ ^]^ and ensures the uniform distribution and stability of charge‐enhancing nanodomains across the film.^[^ ^39^ ^]^

### 2.4 Systematic Engineering of Triboelectric Performance

**Figure 3:**
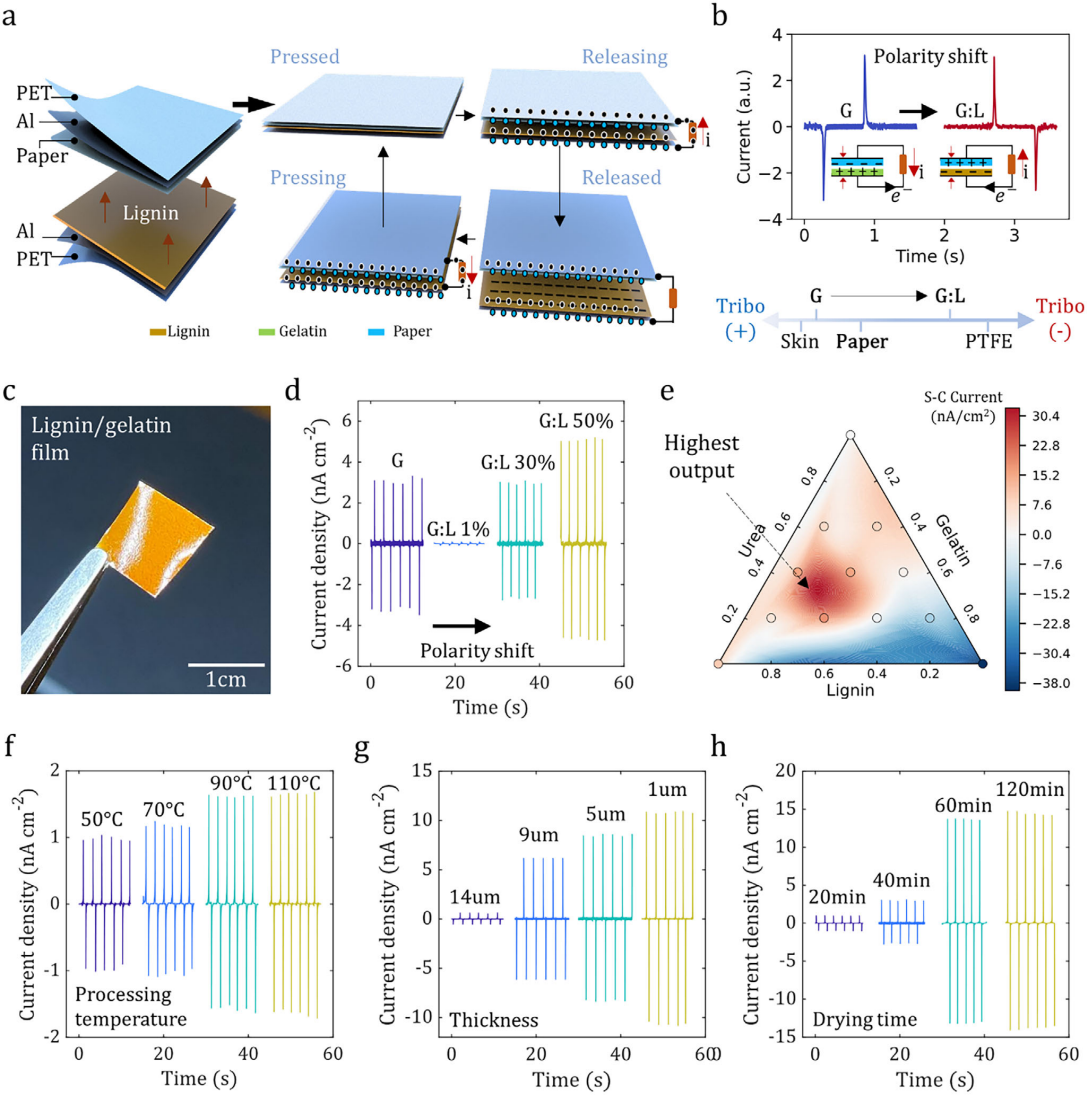
Systematic engineering of material composition and process parameters for peak triboelectric performance. a–c) Schematic of contact‐separation mode device a) and verification of triboelectric polarity inversion. The incorporation of lignin successfully inverted the polarity of the material from tribopositive (pure gelatin) to strongly tribonegative b,c). d,e) Compositional optimization of the triboelectric output. The current density was highly dependent on the lignin content, peaking at 50% (w/w) d). A ternary plot of the current output e) reveals a peak performance at the 30G: 50L:20U ratio, which directly correlates with the optimal ink dispersibility, as shown in Figure 2b. f–h) Optimization of the blade coating process parameters. Systematic variations in the processing temperature (f), film thickness g), and drying time h) revealed optimal conditions of 110 °C, 1 µm, and 120 min, respectively, for maximizing the triboelectric output.

To translate our rationally designed ink into a high‐performance triboelectric material, we systematically engineered its triboelectric properties by configuring a contact‐separation mode device (**Figure** **3**a). We first verified the triboelectric polarity against cellulose (Kimtech), a well‐known tribopositive material with properties similar to those of human skin.^[^ ^40^ ^]^ Characterization confirmed that a pure gelatin film is tribopositive. In contrast, the incorporation of lignin successfully inverted the polarity of the material to strongly tribonegative (Figure 3b), which is a prerequisite for robust triboelectrification against human skin. The triboelectric current output was highly dependent on the lignin content, increasing from nearly zero to a peak of 6 nA cm^−2^ at 50% lignin (Figure 3d). A comprehensive compositional study, visualized in a ternary plot of the current output, revealed peak performance at the 30G: 50L:20U ratio (Figure 3e). This performance map correlates directly with the optimal ink dispersibility (Figure 2b); at concentrations above 50%, the performance degraded because of the decreased dispersibility of lignin and its consequent segregation in the precursor solutions, which reduced the effective contact area between the tribonegative lignin and tribopositive cellulose (Figures S12,S13, Supporting Information).

We further optimized the blade‐coating parameters to maximize the triboelectric output (Figure 3f–h). The performance increased with processing temperature, peaking at 110 °C (Figure 3f). The processing temperature changes the blade coating processability owing to improved fluidity at higher temperatures. The film thickness was also critical, with an optimal thickness of 1 µm yielding a current output of 15 nA cm^−2^. The thinner films were likely limited by air breakdown effects (Figure 3g). A drying time of 120 min was found to be optimal, ensuring complete water removal, as residual moisture is known to decrease tribonegativity^[^ ^41^ ^]^ (Figure 3h). By using urea as a dispersing agent for lignin, the manufacturability of the lignin ink by blade coating was increased (the complete set of all blade‐coated mixtures and their performances are shown in Figures S14–S17, Supporting Information). This systematic process engineering is critical, as it controls the solvent evaporation dynamics that govern the self‐assembly of the functional, charge‐enhancing micro‐nanotexture that increases the effective contact area without additional lithography steps (Figure 2f; Figures S18,S19, Supporting Information).

### 2.5 Optimized Device Performance and Scalability for Manufacturing

**Figure 4:**
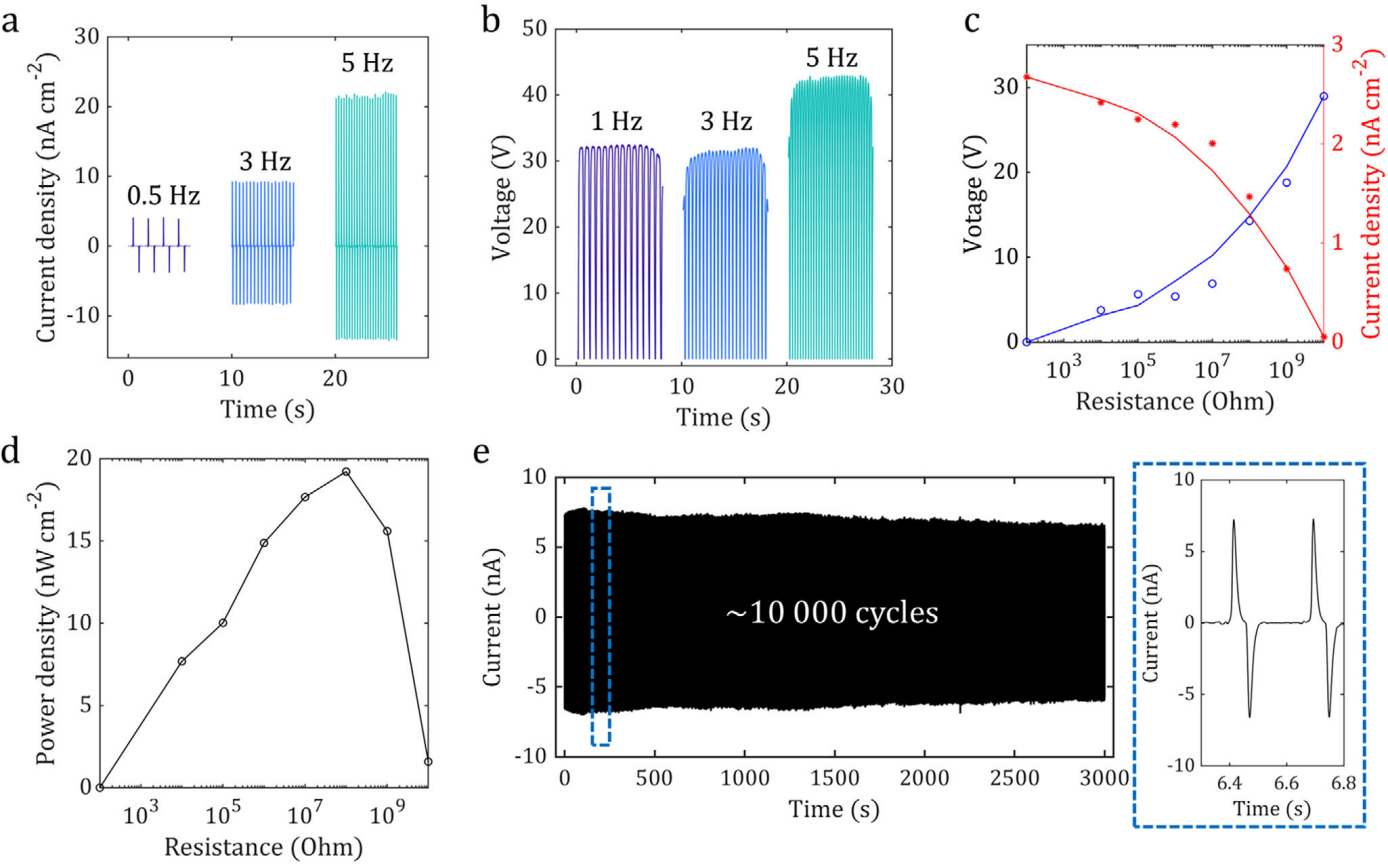
Robust triboelectrical performance and operational durability of optimized lignin‐based device. a,b) Stable open‐circuit voltage (*V*_oc_) exceeding 30 V and short‐circuit current density (*J*_sc_) of 22 nA cm^−^^2^ across a range of operating frequencies. c,d) Electrical output as a function of load resistance, showing a peak power density of 20 nW cm^−2^ at an optimal resistance of 100 MΩ. e) Excellent operational durability, demonstrating stable output with no degradation after 10000 continuous contact‐separation cycles.

The fully optimized film fabricated using the 30G:50L:20U ink and the optimal processing parameters exhibited a stable open‐circuit voltage (*V*_oc_) exceeding 30 V and a short‐circuit current density (*J*_sc_) of 22 nA cm^−2^ across a range of operating frequencies (**Figure** **4**a,b). The device achieved a peak power density of 20 nW cm^−2^ at an optimal load resistance of 100 MΩ (Figure 4c,d) and demonstrated excellent operational durability, maintaining a stable output after 10 000 contact‐separation cycles (Figure 4e). To substantiate the suitability of the ink for industrial‐scale manufacturing, its rheological properties were characterized. The optimized ink exhibited a viscosity of ≈200 mPa·s, which is well within the operational window for scalable roll‐to‐roll coating techniques.^[^ ^42^ ^]^

**Figure 5:**
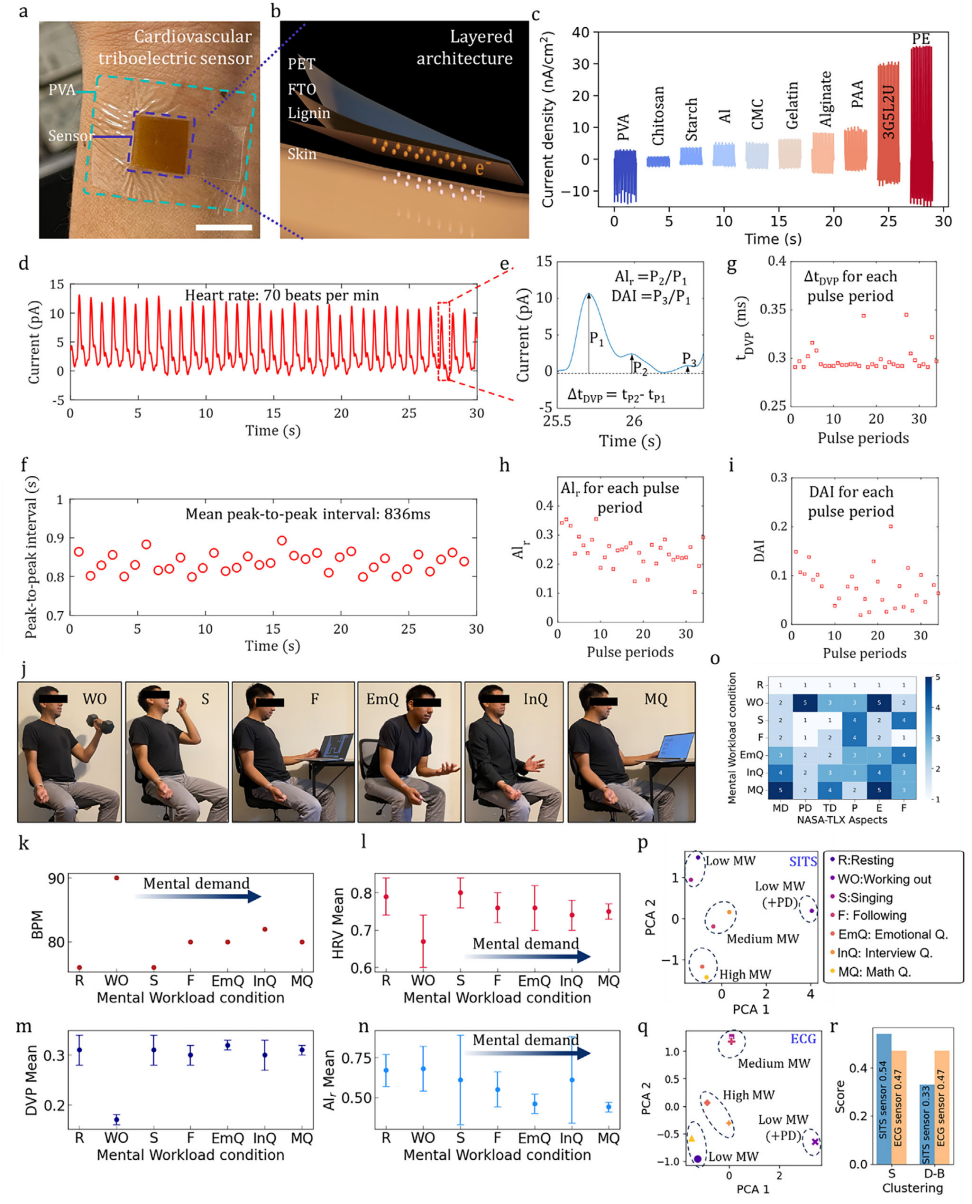
High‐fidelity sensing and objective physiological state classification. a–c) Device architecture and performance validation. Scale bar: 1 cm. The lignin‐SITS a,b) generates a robust electrical output against skin, which is an order of magnitude higher than that of other common biopolymers and comparable to that of synthetic polymers c). d–i) High‐fidelity cardiovascular monitoring. The sensor captured high‐fidelity arterial pulse waves from the wrist d), clearly resolving the characteristic P_1_, P_2_, and P_3_ peaks of the cardiac cycle e). This high signal quality enables the extraction of key cardiovascular parameters, including heart rate variability (HRV) f) and augmentation indices g–i). j–r) Objective classification of mental workload as rigorous proof‐of‐concept validation. Cardiovascular data were recorded during seven distinct tasks j) to elicit different mental workload (MW) states. Principal component analysis (PCA) of four key cardiovascular features k–n) revealed a clear classification of MW states for both lignin‐SITS p) and gold‐standard ECG q). Standard clustering metrics r) quantify this, confirming that the classification performance of the sustainably engineered sensor is comparable to that of a clinical benchmark.

To evaluate the performance of our lignin‐SITS holistically, we benchmarked it against representative biopolymers and synthetic polymer devices across five key axes that are critical for wearable bioelectronics: sustainability, process scalability, biocompatibility, application‐specific efficacy, and electrical output (Table S1, Supporting Information). This comparison reveals that our study resolves a long‐standing trade‐off in the field. While synthetic fluoropolymers, such as PTFE, offer a higher absolute power density, they do so at a significant cost to sustainability and biocompatibility. Conversely, conventional biopolymers have historically struggled to deliver the electrical output required for high‐fidelity sensing, often requiring energy‐intensive processing or the use of complex composites to improve performance. Our work overcomes this critical barrier, achieving an electrical output sufficient for a validated, clinical‐grade sensing application (**Figure** **5**), while establishing a new benchmark for the green, scalable processing of abundant waste biomass. This unique combination of attributes positions our lignin‐based platform as a leading candidate for next‐generation sustainable bioelectronics.

### 2.6 Validation of the Sustainably Engineered Material for Skin‐Integrated Electronics

To validate the functional performance of our sustainably engineered lignin composite, we fabricated it into a skin‐integrated triboelectric sensor (SITS), where the lignin film was in direct contact with the skin (Figure 5a,b). When benchmarked against the skin as a tribo‐counterpart (Figures S20–S22, Supporting Information), the lignin film generated a current output of up to 30 nA cm^−2^. This performance is 4‐ to 6‐fold higher than that of other representative biopolymers, such as alginate and gelatin, and up to 10‐fold higher than that of starch and chitosan, which have been explored for triboelectric sensing.^[^ ^11^, ^43^ ^]^ Critically, this output is comparable to that of the synthetic polymer polyethylene (PE),^[^ ^8^ ^]^ confirming that our sustainably processed material successfully overcomes the historical performance gap in biopolymer‐based electronics for SITS applications (Figure 5c).

### 2.7 High‐Fidelity Cardiovascular Monitoring

The high signal‐to‐noise ratio generated by the lignin SITS enabled continuous, high‐fidelity monitoring of arterial pulse waves from the wrist (Figure 5d). The captured waveform resolved three characteristic peaks of the cardiac cycle: blood ejection (P_1_), reflection from the lower body (P_2_), and reflection from the closed aortic valve (P_3_)^[^ ^44^ ^]^ (Figure 5e). This signal richness allowed for the extraction of key cardiovascular parameters used to diagnose conditions like arterial stiffness, such as the time delay between the first two peaks (Δt_DVP_ = t_P2_‐t_P1_), the radial diastolic augmentation index (DAI) = P_3_/P_1_, and the radial augmentation index AI_r_ = P_2_/P_1_^43^. The measured heart rate was 70 beats/min (BPM), and the mean values for the time delay between peaks (Δt_DVP_ = 298 ms) and radial augmentation index (AI_r_ = 0.244) were typical for a healthy individual.^[^ ^43^, ^44^ ^]^ Studies have shown that a Δt_DVP_ of ≈300 ms is associated with good arterial compliance in healthy individuals. AI_r_ typically increases with age and arterial stiffness. Values below 0.5 are generally considered normal for young to middle‐aged adults.^[^ ^45^ ^]^ The mean peak‐to‐peak interval was 836 ms, and the scatter plot is shown in Figure 5f. Scatter plots of the parameters DAI, Δt_DVP_, and AI_r_ are shown in Figure 5g–i. Heart rate variability (HRV) is the fluctuation in the time interval between adjacent heartbeats. HRV is an important indicator of cardiovascular conditions.^[^ ^43^ ^]^ The measured HRV was 24.7 ms (standard deviation of the peak‐to‐peak interval) (Figure 5f). In our case, HRV was measured in short‐term variability (1 min), and values between 10–50 ms were normal, which is typical of a healthy autonomic response.^[^ ^46^ ^]^ These results demonstrate that the triboelectric signal from our sustainably processed material is sufficiently robust for advanced monitoring of physiological states.

### 2.8 Objective Classification of Mental Workload

As a rigorous test of the sensor's signal quality, we conducted a proof‐of‐concept study to objectively classify the user's mental workload (MW). The cardiovascular data (Figures S23–S29, Supporting Information) were recorded during seven distinct tasks designed to elicit different cognitive and physical loads: resting (R), working out (WO), singing (S), following (F), and answering emotional (EmQ), interview (InQ), and mathematical (MQ) questions (Supplementary Table S2, Supporting Information). We used the six aspects recommended by the subjective NASA‐TLX test, with each aspect ranked from 1 to 6 as recommended.^[^ ^47^ ^]^ The six aspects considered were mental demand (MD), physical demand (PD), temporal demand (TD), performance (P), effort (E), and frustration (F). While the extracted cardiovascular features (BPM, HRV, and AI_r_) correlated with subjective NASA‐TLX survey scores (Figure 5j–o), our goal was to create a fully objective classification model that overcomes the limitations of subjective interpretation.^[^ ^48^ ^]^ Figure 5j–m show the average BPM, DVP, HRV, and AI_r_, where we observed a correlation of the BPM, HRV, and AI_r_ for all conditions (Figure 5n), except for the WO, with the MD aspect score determined by the standard NASA‐TLX survey (Figure 5o). The BPM increased for F, EmQ, InQ, and MQ. In contrast, HRV and AI_r_ decreased for all conditions (Table S3, Supporting Information). Although cardiovascular measurements can assess MW, the perception of MW can vary from person to person, and the NASA‐TLX survey has a subjective interpretation.^[^ ^48^ ^]^ To visualize the inherent separability of the data, principal component analysis (PCA) was performed on the feature space.^[^ ^49^ ^]^ Figure 5p shows the PCA of four cardiovascular parameters (BPM, DVP, HRV, and AI_r_), where we observed a clear classification of these cardiovascular features into four distinct groups: low MW (resting, singing), low MW with physical demand (working out), medium MW (following, emotional questions), and high MW (interview, math questions) (Figure 5p). This objective categorization aligns with established research on cardiovascular sensors for non‐subjective measurements of MW, where PD can influence MW drastically and can be distinct from the cognitive load,^[^ ^50^ ^]^ as seen in the PCA analysis. In addition, resting and singing did not require a high MW, as observed in PCA. On the other hand, questioning tasks, such as interviews and mathematical and psychological tests, are associated with elevated cardiovascular activity and hence medium to high MW.^[^ ^50^ ^]^ These results demonstrate that the triboelectric cardiovascular sensor can provide a non‐subjective association between tasks and MW.

Critically, this classification capability was benchmarked against a gold‐standard ECG sensor (Figure 5p‐r and Figure S30–S33, Supporting Information). Our sensor displayed the same categorized MW groups as the ECG (low MW, low MW with PD, medium MW, and high MW), as shown in Figure S33 (Supporting Information). Figure 5q shows that our lignin‐SITS sensor is as good as the ECG for correlating with the MW conditions. To quantify the quality of this class separation, we calculated the standard clustering metrics. The Silhouette and Davies–Bouldin index scores^[^ ^51^ ^]^ for the SITS‐derived data were comparable to those from the ECG data (Figure 5r), indicating that our sensor produces feature clusters that are as distinct and well defined as the clinical benchmark. This high degree of class separability confirms that the signal from our sustainable sensor is robust enough for use in objective, clinical‐grade assessment of physiological states, overcoming the limitations of subjective surveys,^[^ ^50^ ^]^ such as those from the traditional subjective NASA‐TLX questionnaire (Figure 5o).

## 3 Discussion

This work establishes a comprehensive material‐to‐system pathway for valorizing industrial lignin waste, resolving the long‐standing trade‐off between the performance and sustainability of functional materials. Our central achievement is not merely the creation of a new biopolymer ink, but the demonstration of a chemical engineering design principle, where rational process design transforms an intractable industrial byproduct into a high‐performance electronic material. The foundation of this principle is the process‐as‐design strategy. Guided by a predictive framework based on Hansen solubility parameters, we rationally formulated a benign aqueous ink, creating conditions for thermodynamically driven self‐assembly of lignin polymers. This process directs the spontaneous phase separation of lignin during printing to create a functional charge‐enhancing nanotexture. This unlocks an emergent strong tribonegativity in the formed lignin films, which rivals that of other biopolymers and is comparable to that of synthetic polymers, a feat accomplished while preserving the molecular integrity and inherent biocompatibility of lignin. The weak or unfavorable tribonegativity of biopolymers such as cellulose and chitosan has historically necessitated reliance on unsustainable fluoropolymers for high‐performance skin‐integrated triboelectronics. Our work overcomes this barrier, demonstrating that an industrial biomass byproduct can be engineered into a material whose triboelectrical output against the skin is an order of magnitude higher than that of other common biopolymers and is sufficient for high‐fidelity, clinical‐grade physiological monitoring. All triboelectric tests in this work were carried out under controlled laboratory conditions (22 ± 2 °C, 40–50% RH), under which the lignin films exhibited stable output during both 10000‐cycle durability testing and wrist‐worn monitoring (Figures 4e and 5d–i). In practical devices, the blade‐coated lignin films can be combined with standard breathable encapsulation and medical‐grade adhesive architectures, as used in commercial ECG patches, to further manage the local temperature and humidity without modifying the triboelectric interface with the skin.

As our benchmarking analysis shows, this establishes a new standard in which high sensing functionality is achieved without compromising sustainable sourcing or green processing. The implications of this study extend to a new paradigm in sustainable manufacturing. The aqueous, printable nature of our lignin ink makes it directly compatible with industrial roll‐to‐roll coating techniques, such as gravure and slot‐die coating. This substantiates a viable pathway for the low‐cost, large‐scale production of sustainable electronic materials, directly enabling a circular economy for upcycling the estimated 50–70 million tons of lignin waste generated annually by the pulp and paper industry. More broadly, the design principle established herein, which uses a process to unlock emergent functions in biopolymers, is not limited to lignin. This strategy opens the door to a new class of functional materials derived from other abundant biomass wastes such as chitin and suberin. The ability to create high‐performance materials from such feedstocks can accelerate the development of fully biodegradable, patch‐scale human‐machine interfaces and distributed bioelectronics systems. The high signal‐to‐noise ratio of the triboelectric signal is sufficient to interface with low‐power wireless telemetry systems, thereby opening a direct route for devices that can seamlessly integrate with both the human body and the natural environment.

## 4 Experimental Section

### Materials and Synthesis: Softwood Kraft Lignin Extraction

Softwood kraft lignin was extracted from a pine source. The material was pulped in a Parr 4520 reactor with 10% NaOH solution at 180 °C for 30 min. The resulting black liquor was separated via filtration and centrifugation. Lignin was subsequently precipitated via acidification of the black liquor to a pH of 2.0 using hydrochloric acid. The precipitate was washed twice with purified water and air‐dried overnight to obtain the final lignin powder.

### Materials and Synthesis: Preparation of Aqueous Lignin Ink

Lignin, gelatin, and urea powders were mixed in a 25‐mL flask at the specified weight ratios to a total mass of 800 mg. Distilled water (4 mL) was added, and the mixture was heated to 130 °C on a hot plate while stirring at 300 rpm for 1 h to form a homogenous viscous ink. The ink was cooled to room temperature prior to use.

### Structural and Chemical Characterization: Evaluation of Lignin Dispersibility

The gravimetric dispersibility of lignin in various formulations was evaluated as previously described. After stirring for 1 h at 130 °C, the insoluble residue was separated by centrifugation (5000 rpm, 10 min). The supernatant was decanted, and the residue was filtered, dried at 100 °C for 1 h, and weighed to determine the percentage of non‐dispersed lignin.

### Structural and Chemical Characterization: Hansen Solubility Parameter (HSP) Analysis

The molecular compatibility between the ink components was analyzed using the HSP framework, which partitions the total cohesive energy density (δ_T_) into contributions from dispersion (δ_D_), polar (δ_P_), and hydrogen bonding (δ_H_) forces. The fractional contributions of these parameters were visualized using a Teas graph (Figures S1,S2, Supporting Information) to predict the miscibility.

### Structural and Chemical Characterization: 2D‐HSQC‐NMR Spectroscopy

Nuclear magnetic resonance (NMR) spectra of the raw lignin and lignin‐urea‐gelatin mixture were acquired using a Bruker Avance III 500‐MHz spectrometer with an N_2_ cryo‐platform prodigy probe. ≈50 mg of lignin or the freeze‐dried mixture was suspended in 0.5 mL DMSO‐d_6_ in an NMR tube. The 2D ^13^C‐^1^H heteronuclear single quantum coherence (HSQC) experiments were carried out with a Bruker pulse sequence (hsqcetgpspsi2.2) on an N_2_ cryoprobe (BBO 1H&19F‐5 mm) with the following acquisition parameters: spectral width 12 ppm in F2 (^1^H) dimension with 1024 data points (acquisition time 85.2 ms), 200 ppm in F1 (^13^C) dimension with 256 increments (acquisition time 5.1 ms), a 1.0‐s delay, a ^1^J_C‐H_ of 145 Hz, and 256 scans. The acquired spectra were processed using Bruker Topspin 4.0 (Mac) software. The central DMSO solvent peak (δ_C_/δ_H_ at 39.5/2.49) was used for chemical shift calibration. The assignments of the lignin compositional subunits and inter‐unit linkages were based on the contours reported in the HSQC spectra.^[^ ^52^ ^]^ Semi‐quantitative analysis of the HSQC cross‐signal intensities was performed to measure the S, G, and side chain linkages. The C_α_ signals were used for contour integration for interunit linkage estimation.

### Structural and Chemical Characterization: Microscopy (AFM, KPFM, Fluorescence, SEM)

The film morphology was characterized using atomic force microscopy (AFM) (Keysight 5500) in the tapping mode with a silicon cantilever (ACTA, AppNano). The surface potential was simultaneously measured using Kelvin probe force microscopy (KPFM) with a gold‐coated silicon cantilever (ACTGG, AppNano). Fluorescence microscopy was performed using a WITec AFM/Raman device at an excitation wavelength of 632.8 nm. Scanning electron microscopy (FE‐SEM) was performed using a Hitachi SU8230 instrument at varying voltages (0.8, 3, and 6 kV) without sputtering.

### Structural and Chemical Characterization: ATR‐FTIR Spectroscopy

Attenuated total reflection Fourier‐transform infrared spectroscopy (ATR‐FTIR) was performed with a Thermo Nicolet Nexus FTIR spectrometer, averaging 8 scans at a resolution of 4 cm^−1^ in the range of 4000 to 800 cm^−1^.

### Device Fabrication and Performance Characterization: Fabrication of the Triboelectric Sensor

Triboelectric devices were fabricated by cutting lignin/Al films to a size of 1 cm^2^. The films were attached to a glass substrate for mechanical support, and copper lead was attached to the Al film for electrical characterization.

### Device Fabrication and Performance Characterization: Triboelectric Characterization

The electrical output was measured by applying a programmed external force using a linear motor (LinMot PS01‐23 × 80) with an operating distance of 20 mm. The thickness of the films was measured using a mechanical micrometer. The output voltages were measured using Keithley 6514, and the current outputs were measured using a low‐noise current preamplifier (Stanford Research Systems SR570).

### Proof‐of‐Concept Physiological Monitoring Study: Sensor Preparation and Placement

For cardiovascular monitoring, the lignin/Al films were coated onto PVA paper and attached to the subject's wrist using skin as the tribopositive material.

### Proof‐of‐Concept Physiological Monitoring Study: Human Subject Protocol

A proof‐of‐concept study was conducted with one healthy male subject (age 33 years) in compliance with protocols approved by the Institutional Review Board of Purdue University (IRB‐2019‐156). The participant provided written informed consent prior to participation.

## Conflict of Interest

The authors declare no conflict of interest.

## Author Contributions

W.Z.W. conceived the idea; W.Z.W. and R.C. designed the experiments; Y.Z. helped in the isolation and characterization of lignin derived from pine; R.C. conducted the experiments on lignin dispersion; M.L. performed the NMR characterization and analysis; R.C. fabricated and characterized lignin films; R.C. characterized the fluorescence, AFM, KPFM, and FTIR spectra of the lignin films; R.C. fabricated and characterized the cardiovascular triboelectric sensor; R.C. and W.Z.W. analyzed the data; W.Z.W. and R.C. wrote the paper. All authors discussed the results and commented on this paper.

## Supporting information



Supporting Information

## Data Availability

The data that support the findings of this study are available from the corresponding author upon reasonable request.
