# Supplementary material for: Aqueously Upcycled Lignin with Emergent Tribonegativity for Skin‐Integrated Triboelectronics
Source: Adv Mater. 2025 Dec 9;38(9):e18412. doi: 10.1002/adma.202518412 (PMC12902614; doi:10.1002/adma.202518412)
Supplement: Supplementary file 1 — Supporting Information [file ADMA-38-e18412-s001.docx]

Supplementary Information

**Aqueously upcycled lignin with emergent tribonegativity for skin-integrated triboelectronics**

Robert Ccorahua-Santo^1,2^, Mi Li^3^, Yi Zheng^4^*, Wenzhuo Wu^1,2,5,6,7,8,9^*

^1^ Edwardson School of Industrial Engineering, Purdue University, West Lafayette, IN 47907, USA

^2^ Flex Laboratory, Purdue University, West Lafayette, IN 47907, USA

^3^ Center for Renewable Carbon, School of Natural Resources, University of Tennessee, Knoxville 2506 Jacob Drive, Knoxville, TN. 37996-4570 USA

^4^ Department of Grain Science and Industry, Kansas State University, 101C BIVAP, 1980 Kimball Avenue, Manhattan, KS 66506, USA

^5^ Women's Global Health Institute, Purdue University, West Lafayette, IN 47907, USA

^6^ Purdue Institute for Integrative Neuroscience, Purdue University, West Lafayette, IN 47907, USA

^7^ Purdue Institute of Inflammation, Immunology, and Infectious Disease, Purdue University, West Lafayette, IN 47907, USA

^8^ Purdue Institute for Drug Discovery, Purdue University, West Lafayette, IN 47907, USA

^9^ Institute for Physical AI, Purdue University, West Lafayette, IN 47907, USA

* Corresponding authors: W. Z. Wu ([wenzhuowu@purdue.edu](mailto:wenzhuowu@purdue.edu)); Y. Zheng ([yizheng21@outlook.com](mailto:yizheng21@outlook.com))

**Hansen Solubility Parameters calculations**


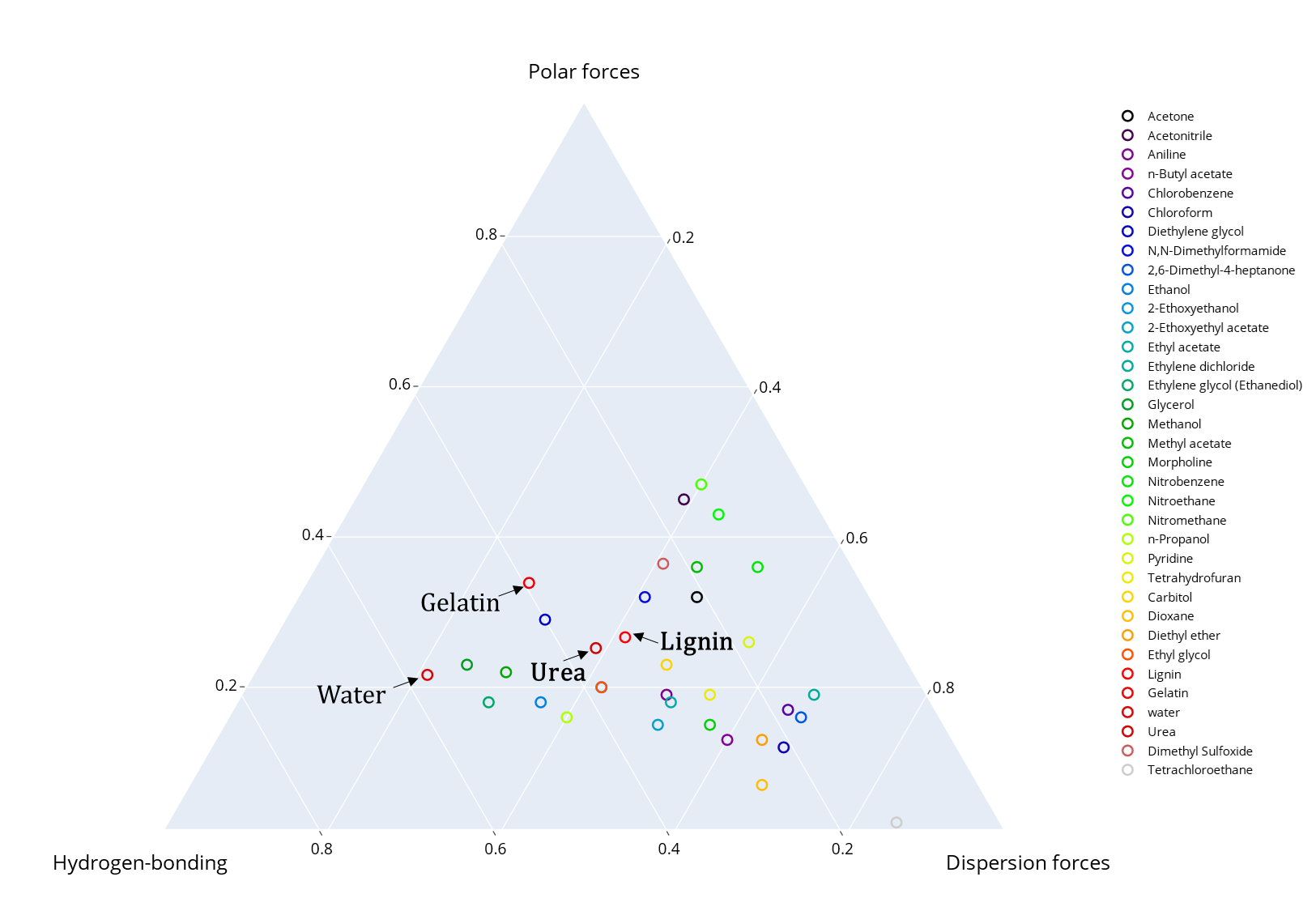


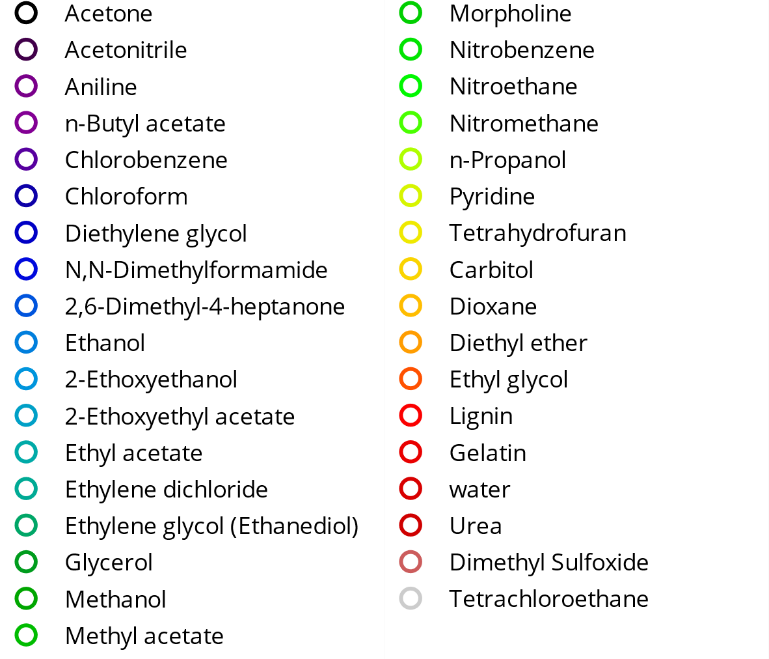


**Figure S1**. **Teas graph of the Hansen Solubility Parameters (HSP) of lignin, urea, gelatin, water, and several organic solvents.** Teas graph shows the representation of the mathematical fractional contributions of δ_T_ and the HSP. The proximity of all components of the lignin ink is shown. Lignin and urea are the closest molecules in this plot.


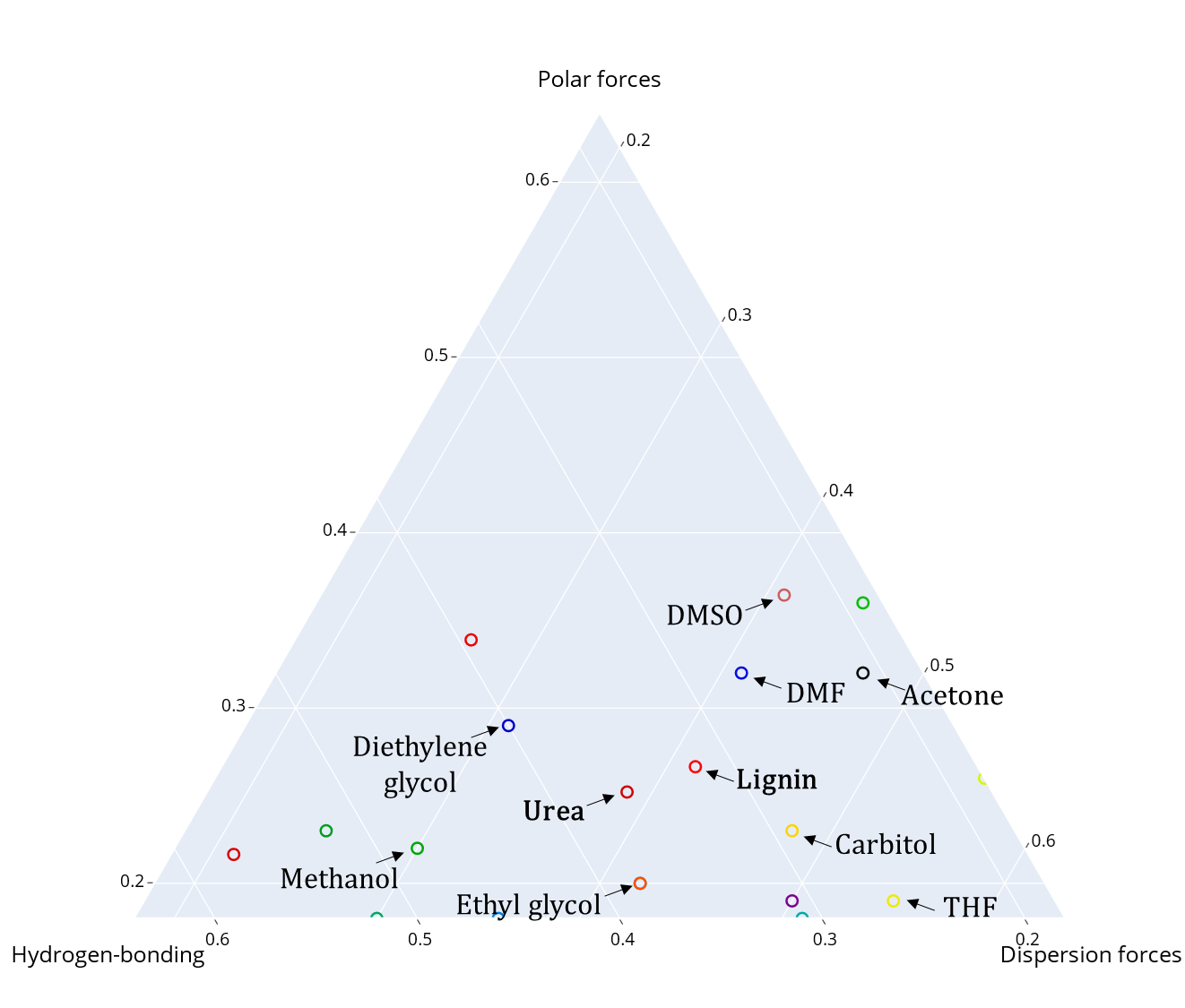


**Figure S2**. **A section (0.18-0.64 for all axis) of the Teas graph shown in Fig. S1.** The fractional contributions of the HSP of common lignin solvents (DMSO, DMF, THF, acetone, and others) are pointed by arrows. The proximity of all common solvents of the lignin ink and urea indicates that urea is the closest molecule to lignin.


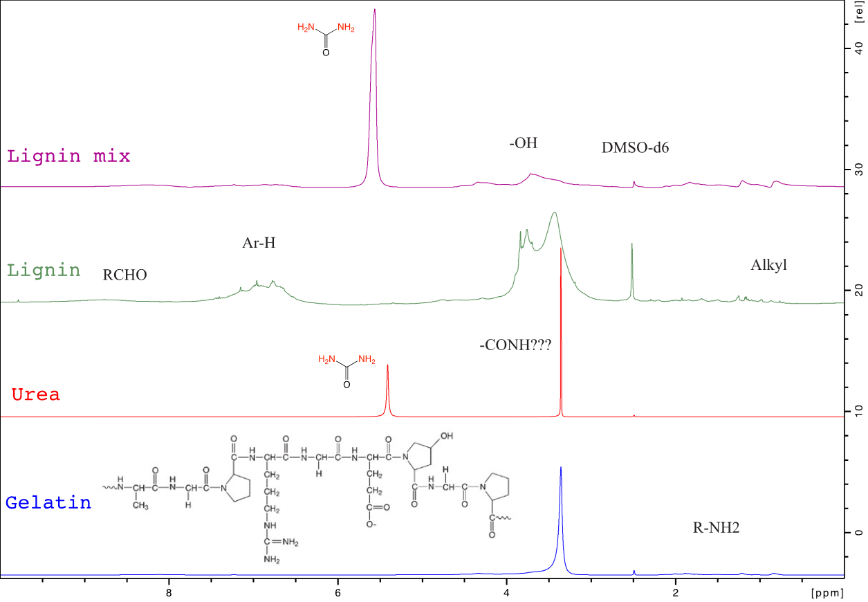


**Figure S3**. **1H-NMR verification of the presence of lignin, urea, and gelatin in the lignin ink.** In NMR spectroscopy, the chemical shift (ppm) quantitatively indicates how shielded or deshielded a nucleus is: High ppm (downfield): Values above ~6 ppm for ¹H or above ~100 ppm for ¹³C suggest a deshielded environment. This often occurs near electronegative atoms or aromatic rings, where electron density is pulled away from the nucleus. Low ppm (upfield): Values below ~3 ppm for ¹H or below ~50 ppm for ¹³C indicate a shielded environment. This is typical in simple aliphatic chains, where electron density is higher around the nucleus, reducing its exposure to the magnetic field.


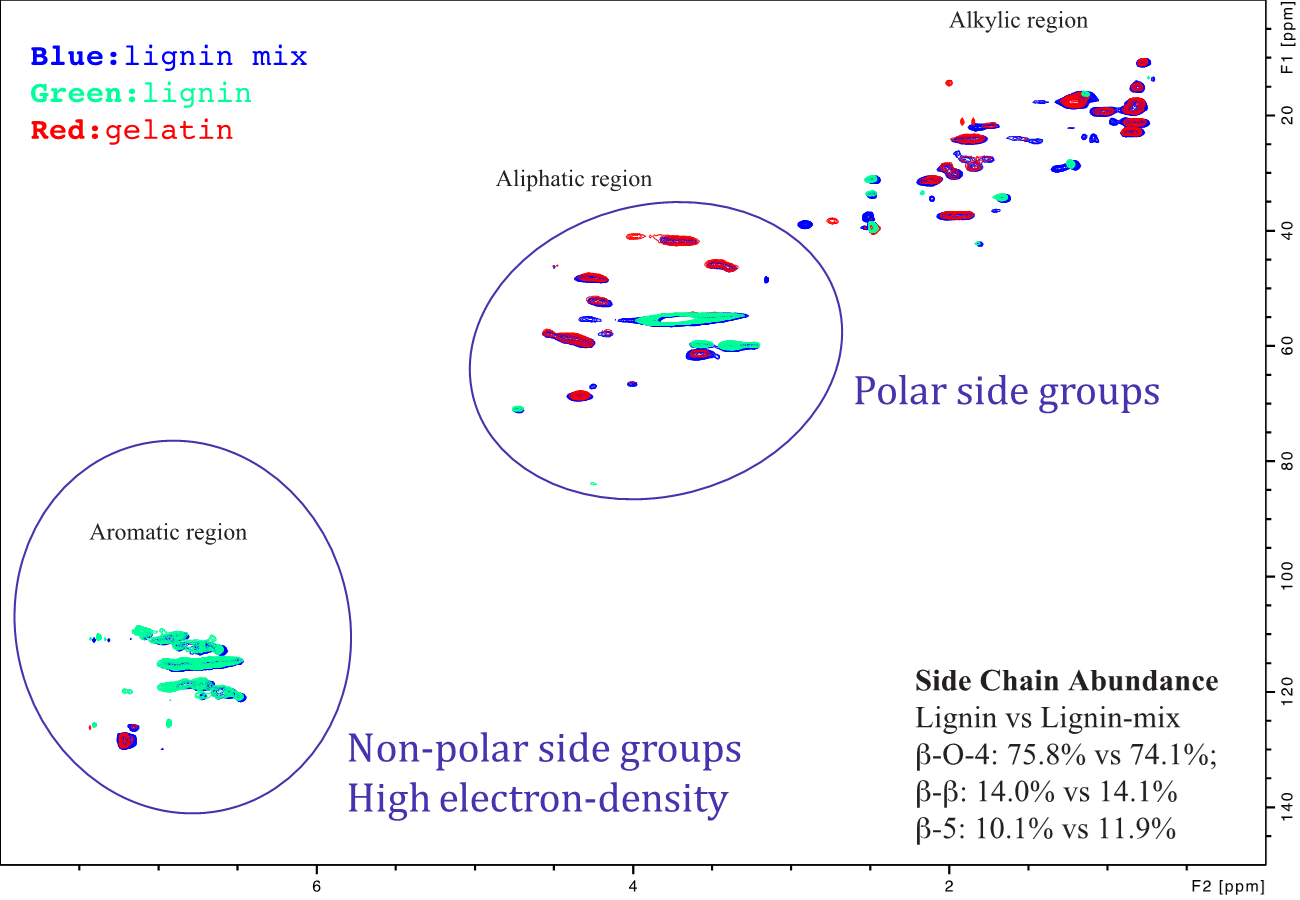


**Figure S4**. **HSCQ-NMR of lignin mixture (blue), pure lignin (green), and gelatin (red).** The HSQC spectrum displays correlations between protons (¹H) and carbons (¹³C) that are directly bonded to each other. In an HSQC spectrum: X-Axis (¹³C): Represents the chemical shifts of carbon atoms. Y-Axis (¹H): Represents the chemical shifts of hydrogen atoms. Cross Peaks: Each spot (cross peak) on the 2D plot indicates a correlation between a proton and its directly attached carbon atom, providing insight into the molecular structure. The provided figure is an HSQC NMR plot that compares the molecular structure of three different substances: lignin mix (blue), lignin (green), and gelatin (red). The key features of the figure are: Aromatic Region (Bottom left): Shows correlations related to aromatic rings, which are likely present in lignin. This region has higher electron density, often associated with non-polar side groups. Aliphatic Region (Center): Displays peaks corresponding to aliphatic chains (non-aromatic hydrocarbons) which are likely involved in polar side groups. Allylic Region (Top right): Contains peaks associated with allylic carbons, often found in the structure of lignin. Color Coding: Blue (Lignin Mix): Represents the HSQC peaks of the lignin mix, indicating how its molecular structure differs or overlaps with pure lignin and gelatin. Green (Lignin): Represents pure lignin, showing its structural characteristics, particularly in the aromatic and aliphatic regions. Red (Gelatin): Represents gelatin, which has a different structure, with distinct peaks in the aliphatic and allylic regions. Side chain abundance: The side chain abundance data compare the relative percentages of specific linkages or side chains (β-O-4, β-β, and β-5) between lignin and the lignin mix. For instance, the β-O-4 linkage is slightly more abundant in pure lignin (75.8%) compared to the lignin mix (74.1%).


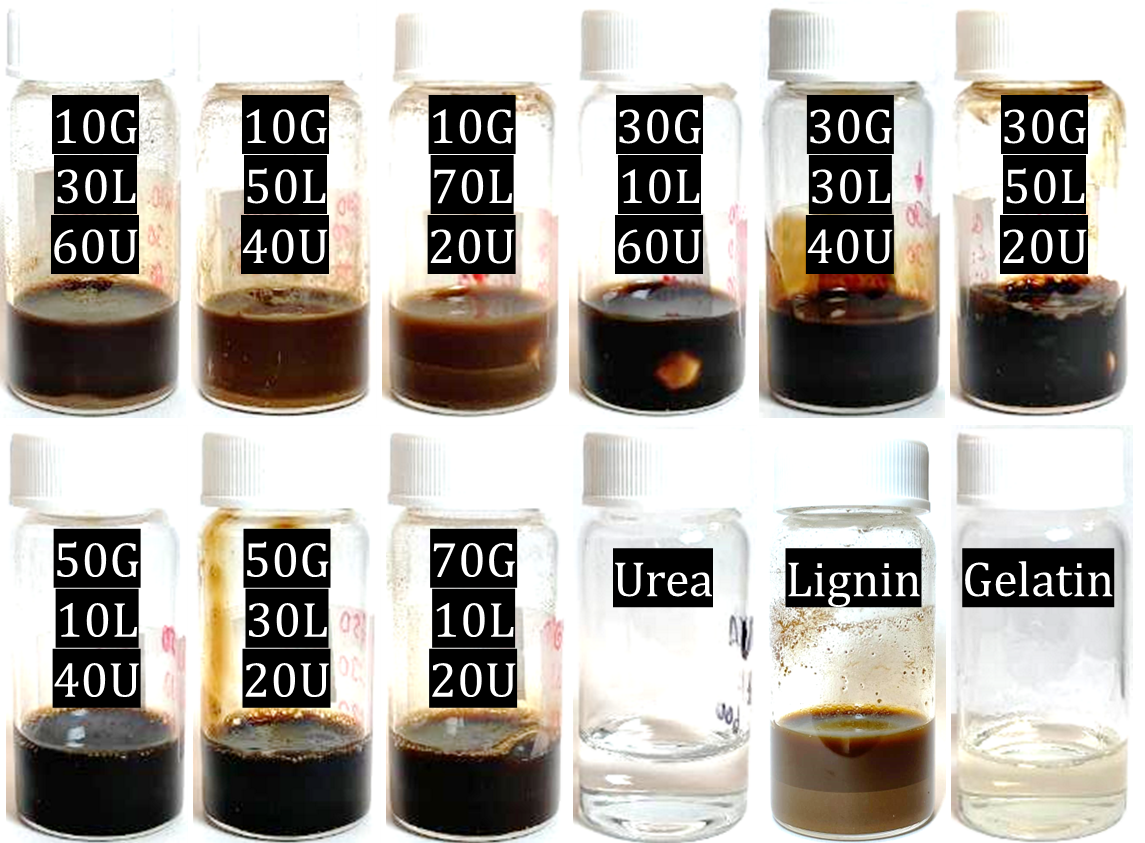


**Figure S5**. **Solutions of lignin, gelatin, and urea at various mixtures ratios. G: gelatin, L: Lignin, U: Urea.**


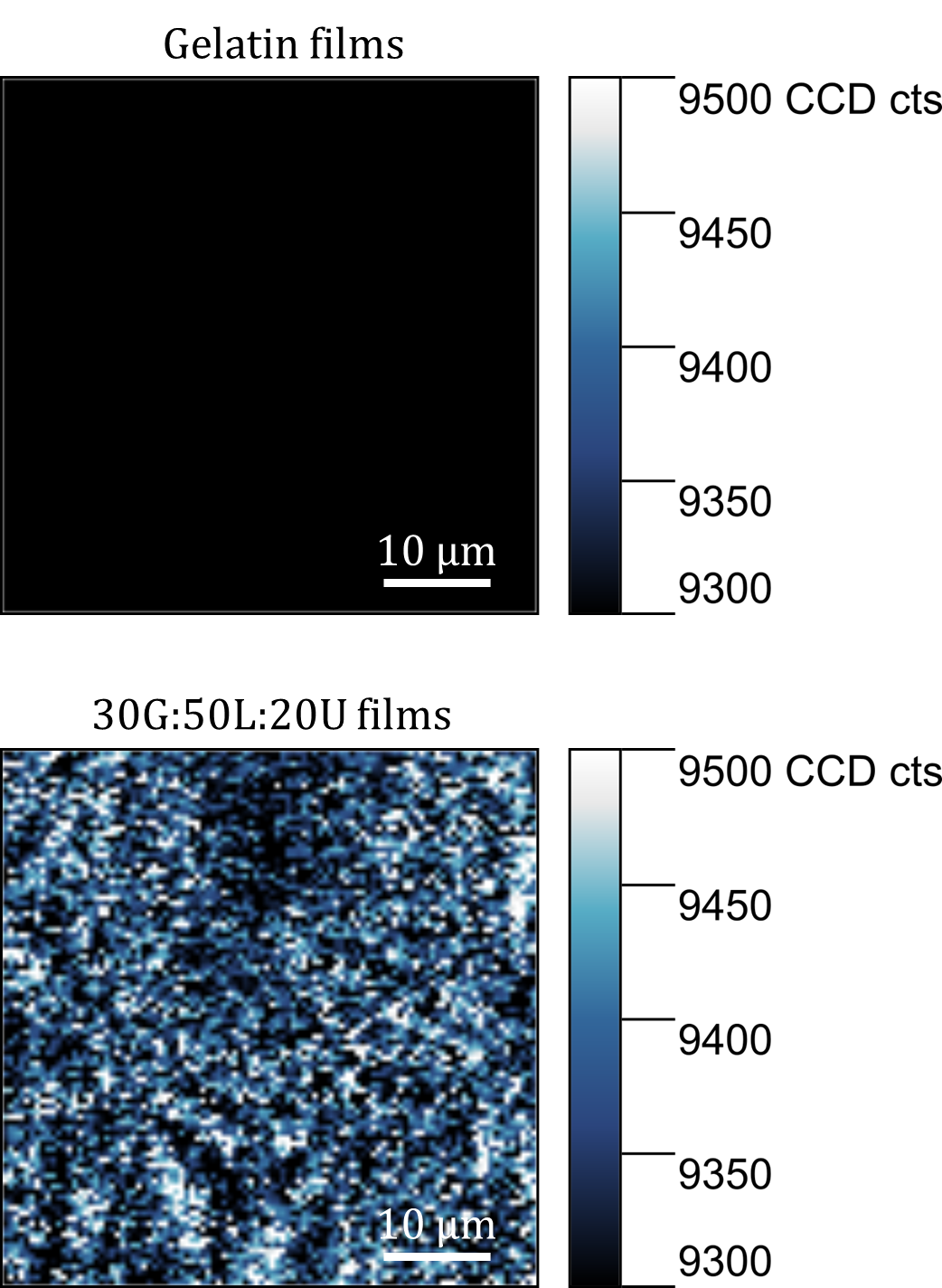


**Figure S6**. **Fluorescence comparison between gelatin and 30G:50L:20U films.**


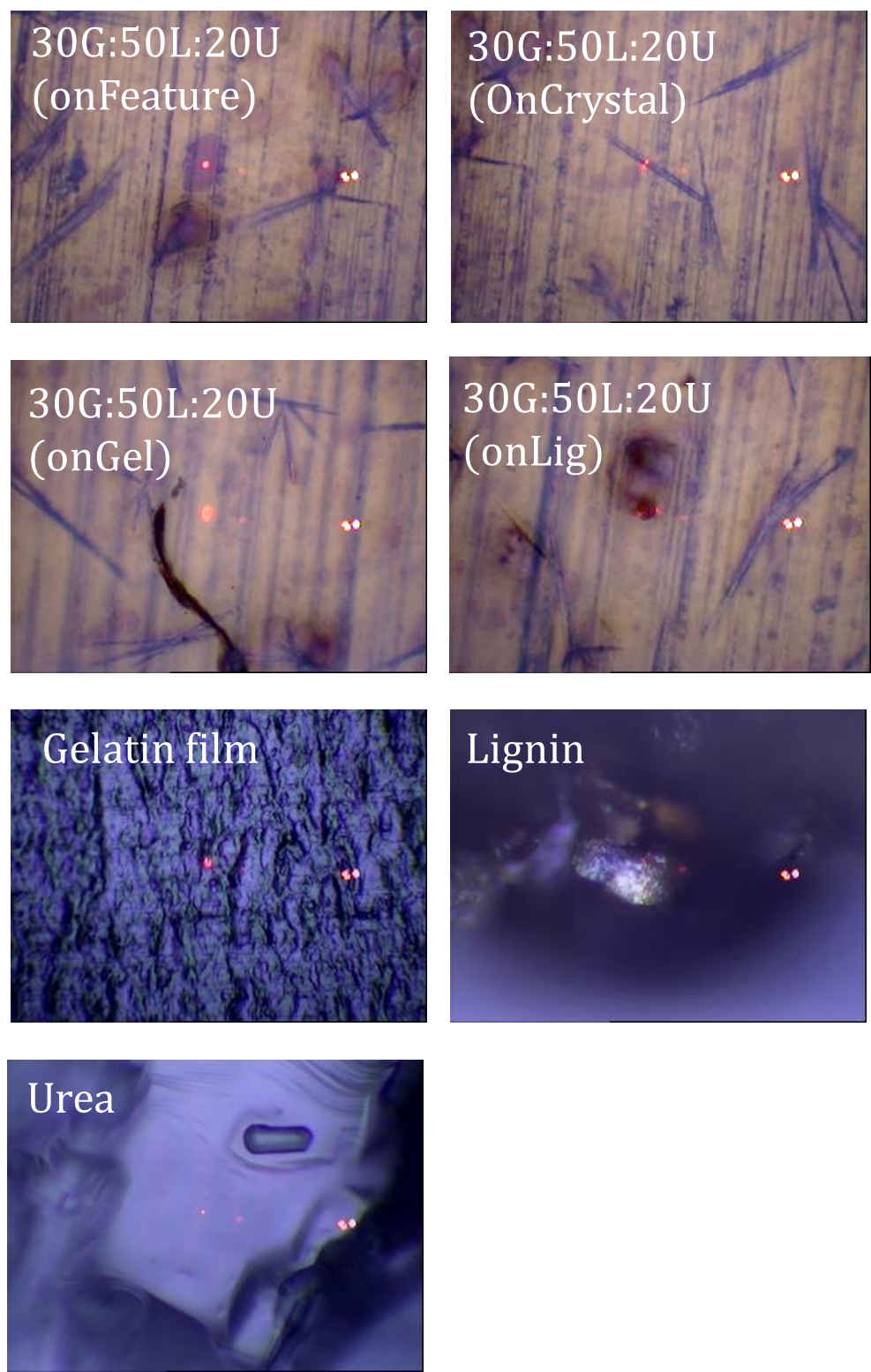


**Figure S7** **Microscopy optical images of the laser position for fluorescence essays of various features on lignin films. G: gelatin, L: Lignin, and U: Urea.**


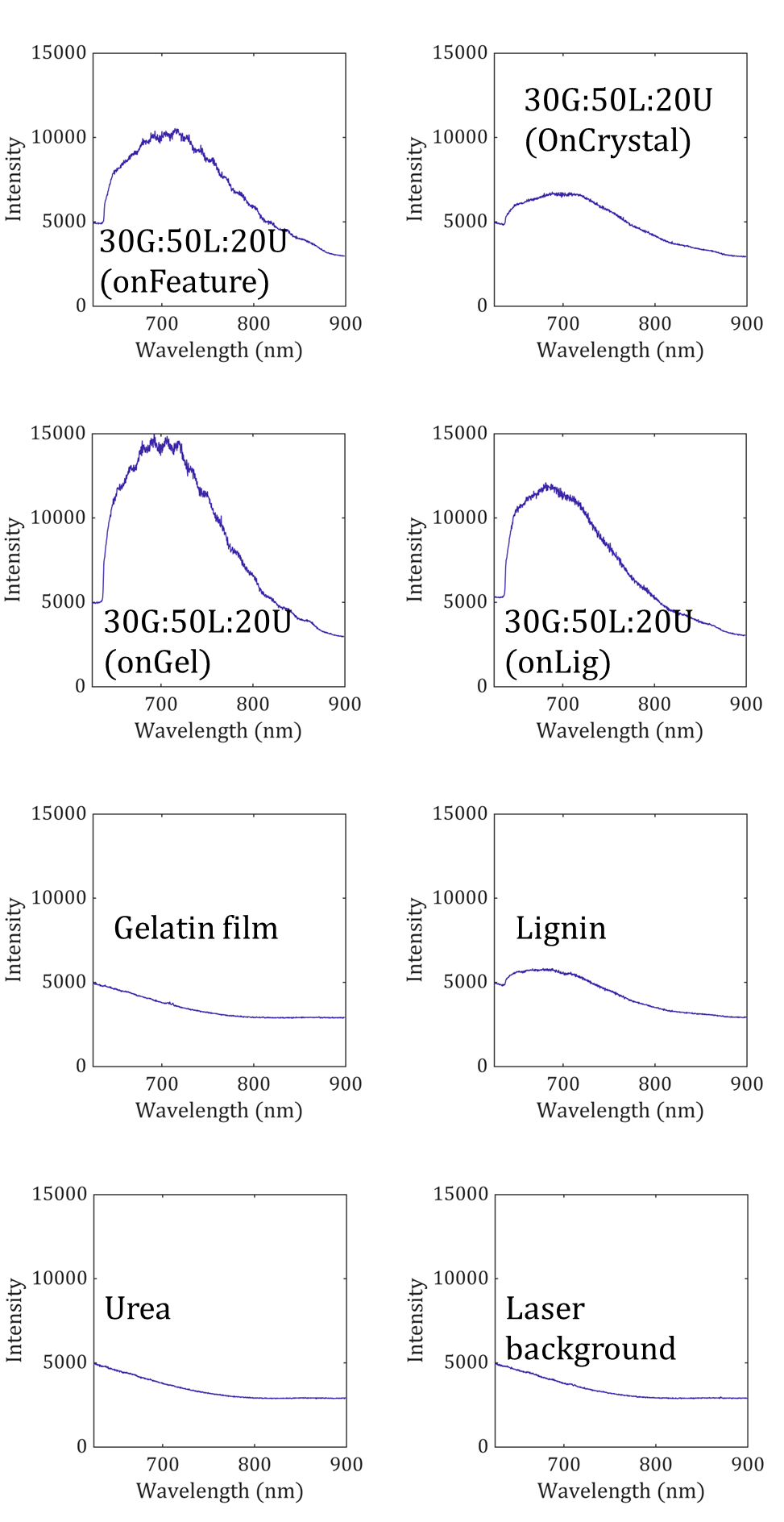


**Figure S8**. **Fluorescence spectra of various features on lignin films. G: gelatin, L: Lignin, and U: Urea.**


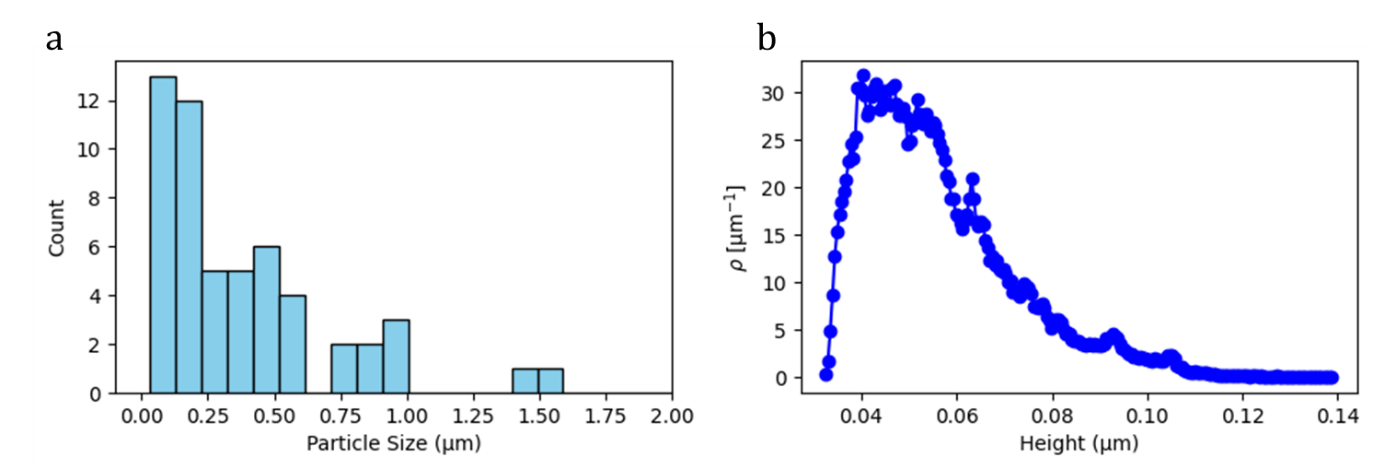


**Figure S9**. (a) Distribution of particle sizes and (b) height distribution in the topographic images of the sample 30G:50L:20U.


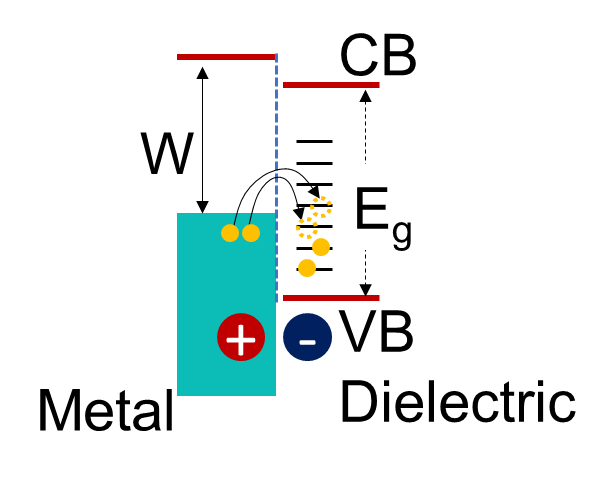


**Figure S10**. **Surface potential measurement by KPFM.** During the characterization, the metallic part is gold, and the dielectric are the dielectric films.


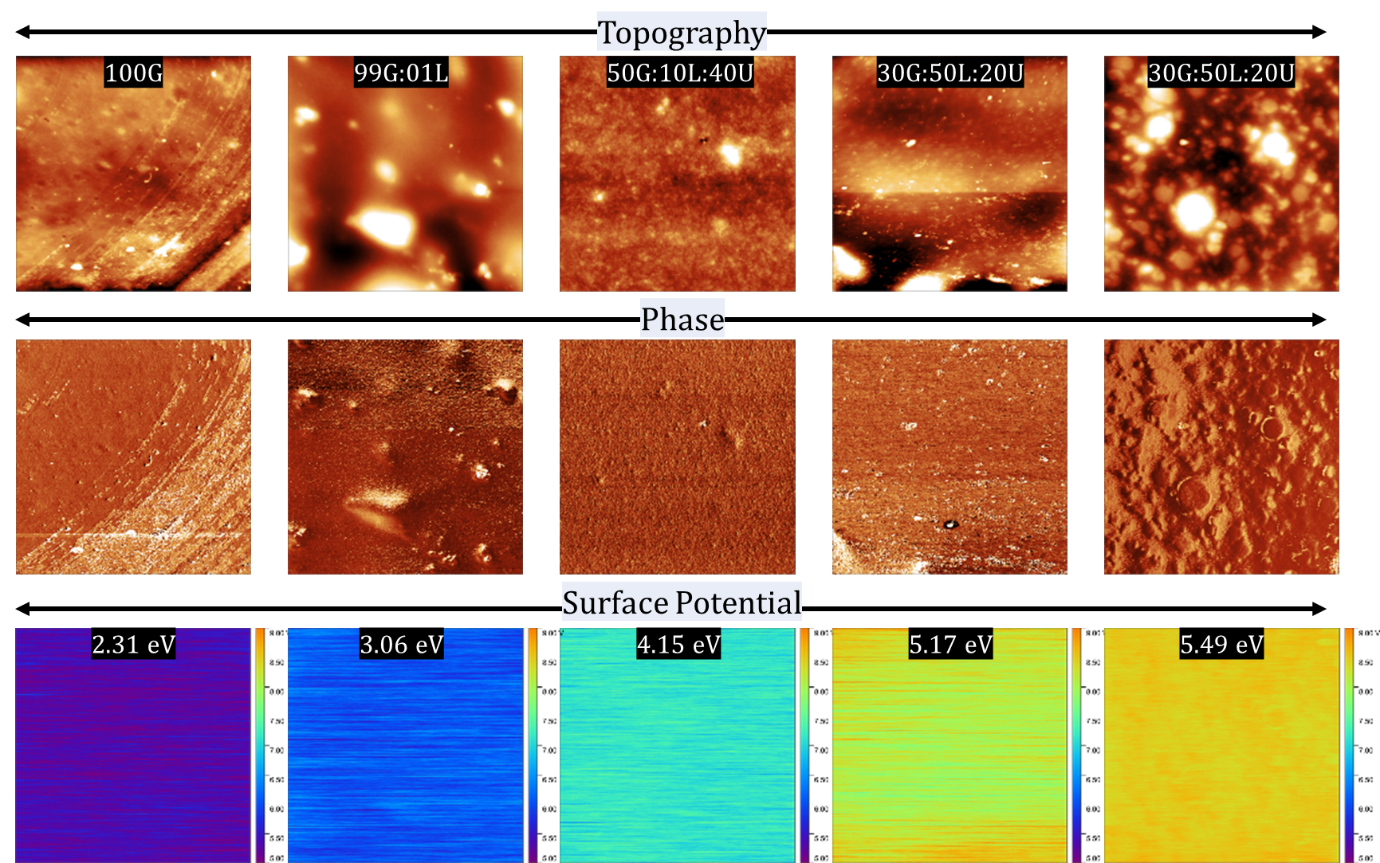


**Figure S11**. **Correlation of topographic, phase, and surface potential KPFM images.** The surface potential is at the same scale for all the mixtures. G: gelatin, L: Lignin, U: Urea, and eV: electron-volts.


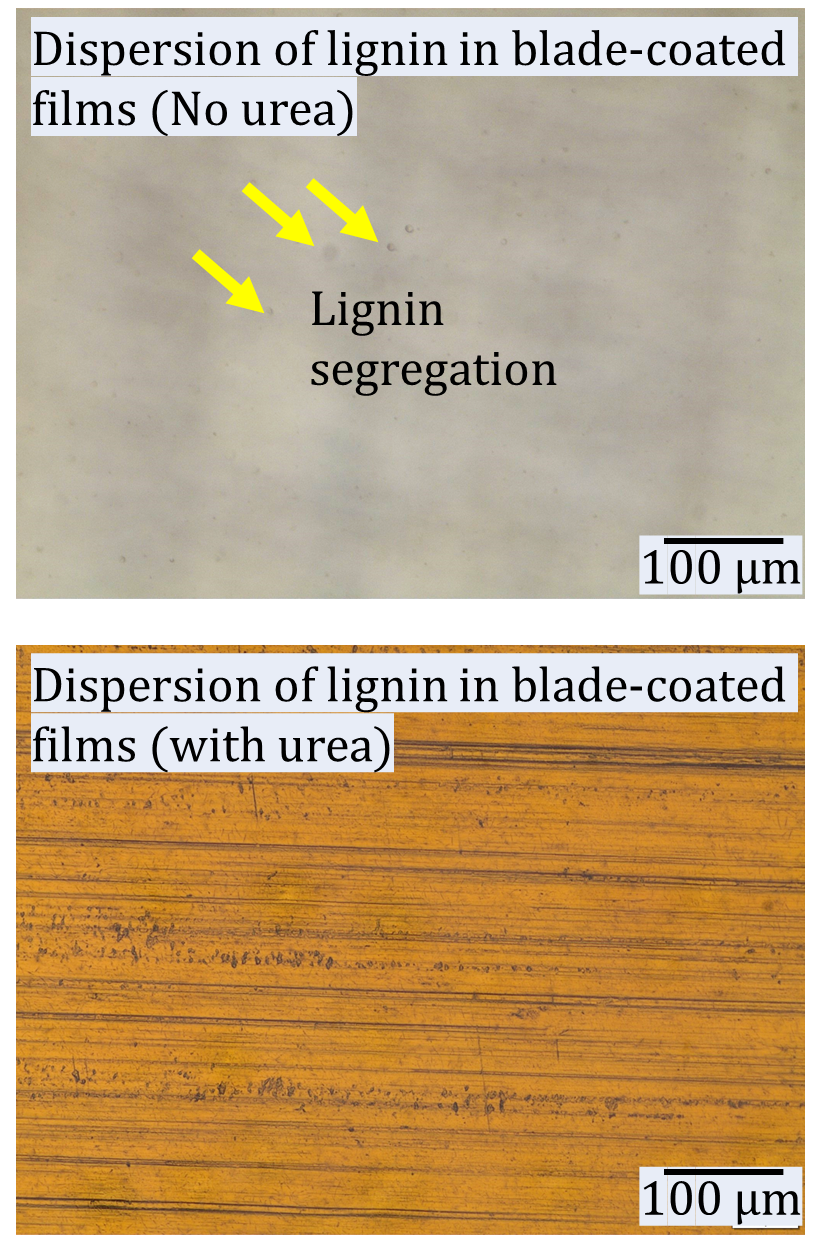


**Figure S12**. **Optical microscope images showing the segregation and dispersion of lignin in blade-coated films.**


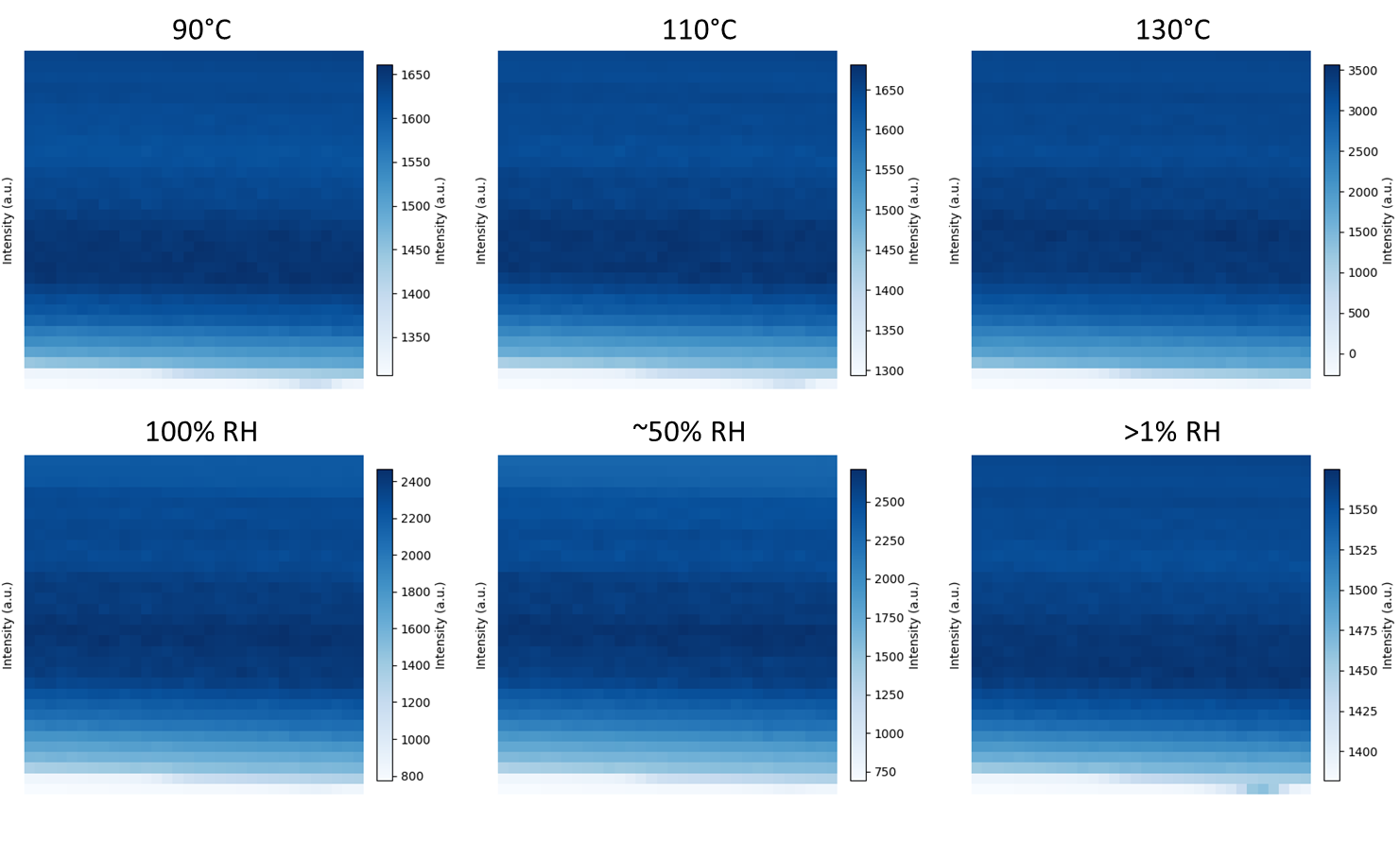


**Figure S13**. Fluorescence images of 30G50L20U films processed at 90, 110 and 130°C, and films stored after films processing at 100%, 50% and below 1% of Relative humidity.

Segregation of lignin in the 30G50L20U films under different temperatures and humidities:

The segregation of lignin during drying is reproducible. Fluorescence maps (Figure SN) show comparable intensity gradients, with an intensity average from 650 to 800 nm, for films processed at 90, 110, and 130 °C, indicating that lignin domains form consistently even after drying at different temperatures.

Environmental exposure tests confirm that films stored at 100%, ~50% RH and <1% RH retain identical homogeneous lignin distribution. Thus, the film formation and lignin segregation process are robust and repeatable when drying parameters vary and when storage is moisture-saturation level.


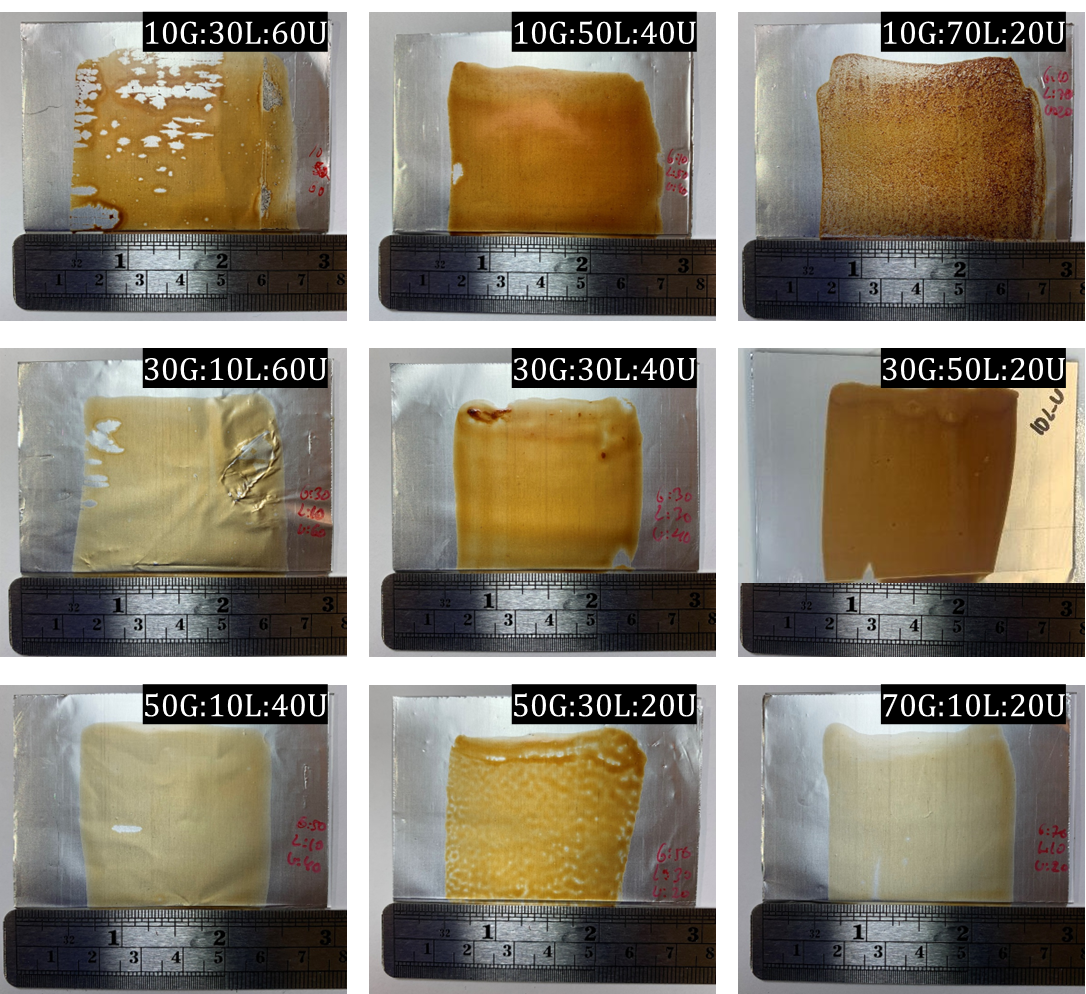


**Figure S14**. **Blade-coated lignin films on Al substrates at various mixture ratios.** G: gelatin, L: Lignin, and U: Urea.


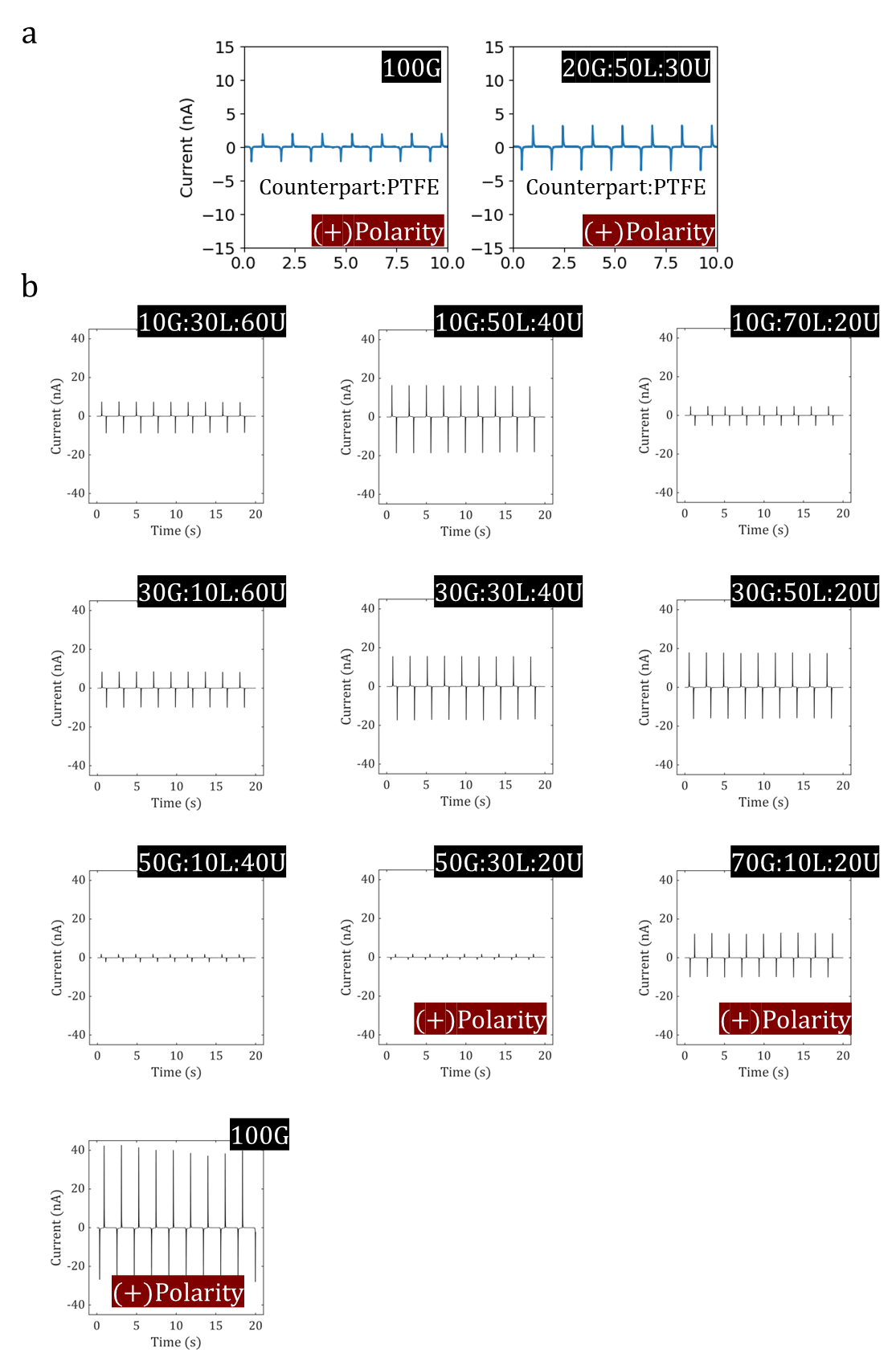


**Figure S15**. **Current TENG performance of blade-coated lignin-based films**. (a) PTFE and (b) cellulose as a counterpart. G: gelatin, L: Lignin, and U: Urea.


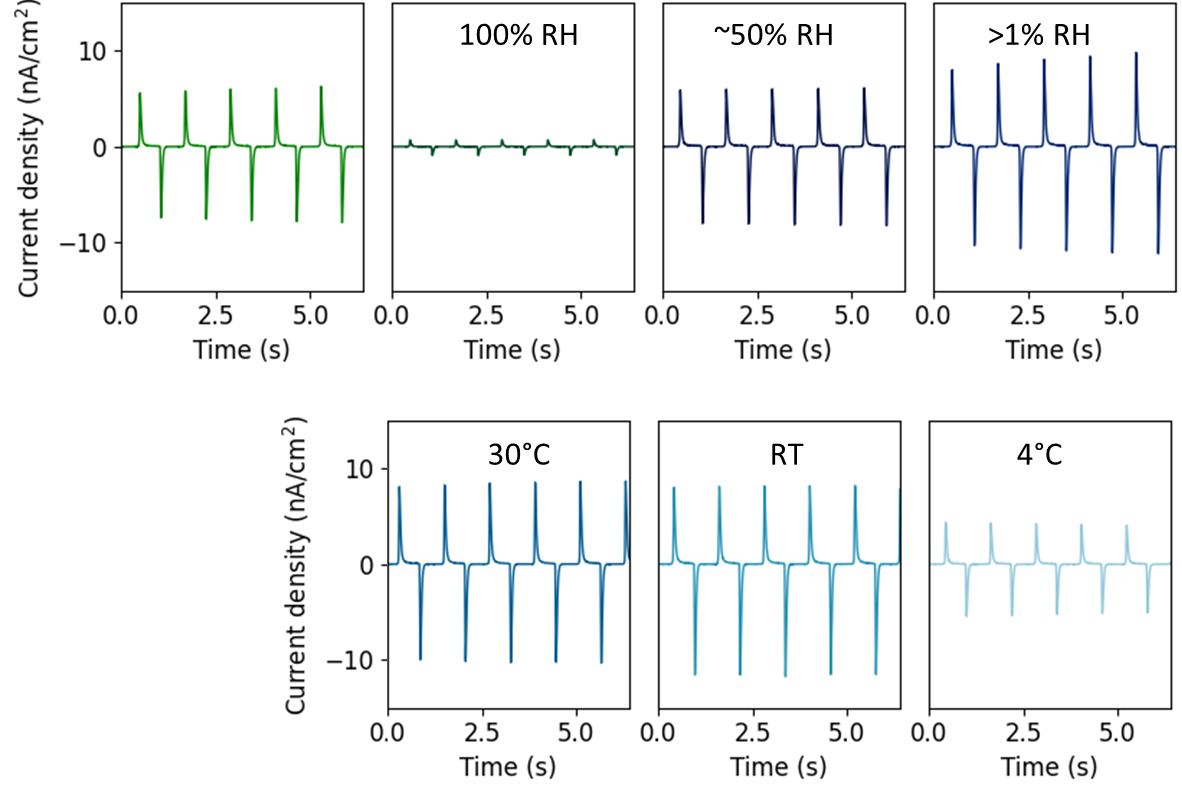


**Figure S16**. Current density of 30G50L20U films after 24h exposed to environments of 100, 50 and 1% of relative humidity and temperatures from 30 to 4°C.

The triboelectric performance of 30G50L20U films correlates directly with the exposition to different humidities and temperatures. Under <1% RH and ~50% RH, the current density remains stable at ±10 nA cm-2. In contrast, 100% RH suppresses the signal by 80% due to water-induced charge screening. Temperature variations between 30 °C, RT, and 4 °C cause <50% amplitude change, confirming that environmental moisture is the primary factor influencing reproducibility. The triboelectric output is strongly influenced by environmental moisture, both from ambient humidity and condensed water at low temperature. This reduction is attributed to the hygroscopic nature of gelatin, which absorbs water and increases the dielectric loss, screening surface charges and reducing effective potential differences during contact–separation.

Temperature-dependent behavior follows the same mechanism: at 4 °C, condensed moisture elevates interfacial conductivity, partially neutralizing accumulated charges. In contrast, 30 °C and RT maintain nearly identical output profiles, confirming that humidity rather than thermal energy governs reproducibility. Hence, controlling environmental water content is essential to ensure consistent triboelectric performance across batches.


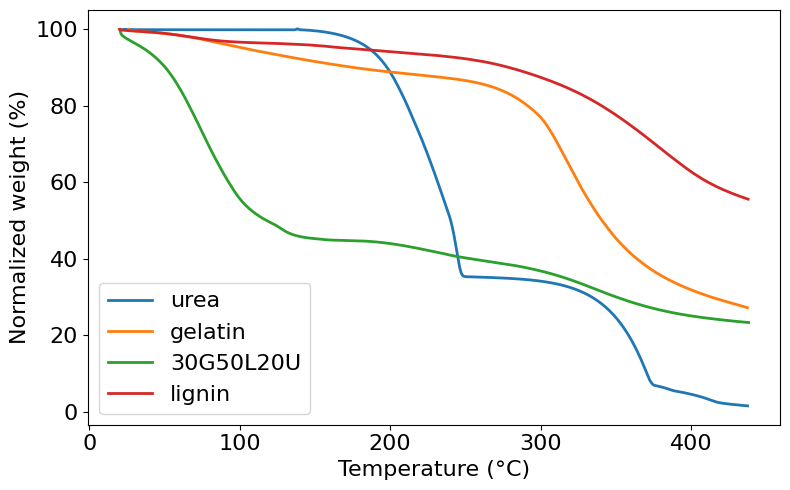


**Figure S17**. Thermogravimetric curve of urea, gelatin, lignin and sample 30G50L20U.

The weight loss of the sample 30G50L20U until 130°C is due to humidity loss and first and second hydration layers of water surrounding molecules of gelatin, urea and lignin, respectively. No decomposition of urea is observed until after 160°C, confirming that the films 30G50L20U have no urea degradation when drying at 130°C.


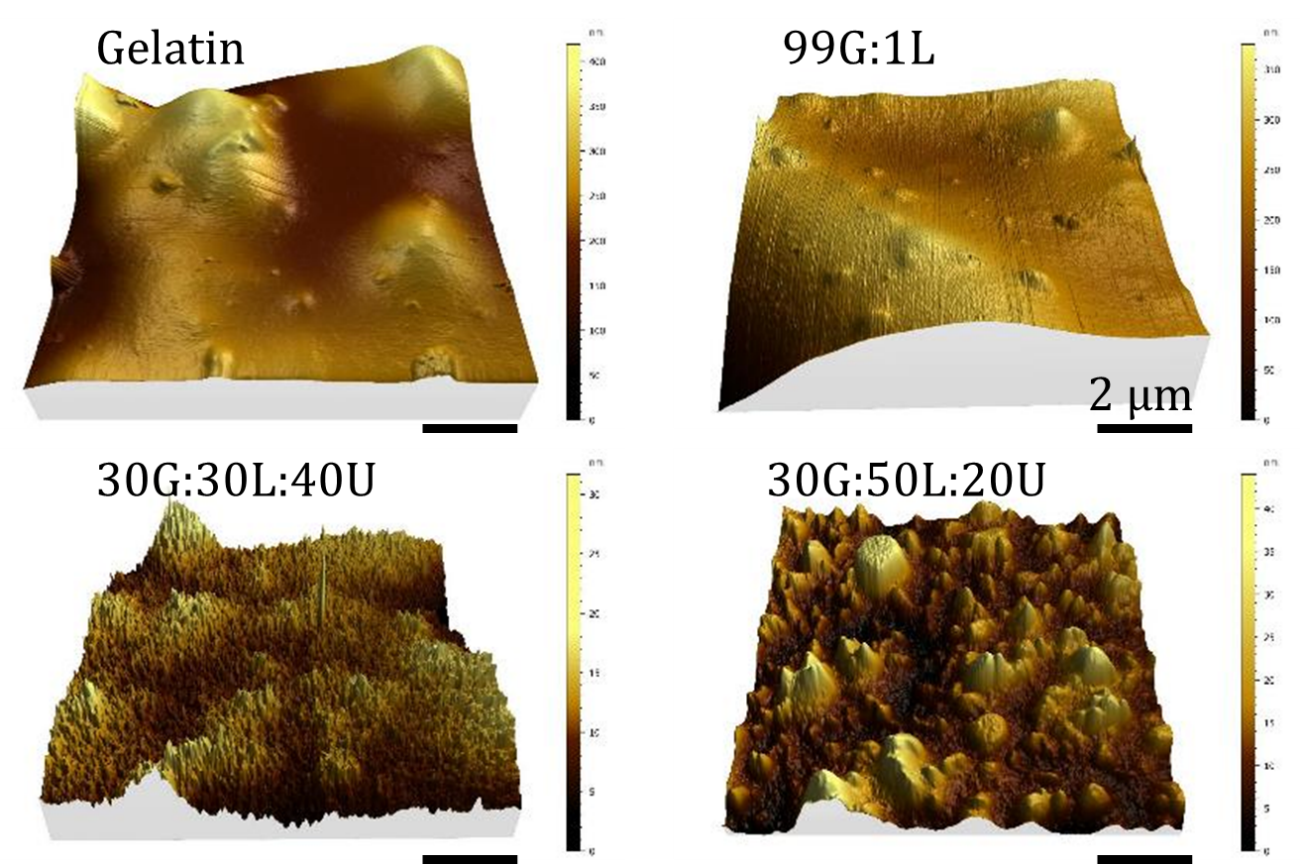


**Figure S18**. **AFM topographic images and roughness correlation.** G: gelatin, L: Lignin, and U: Urea.


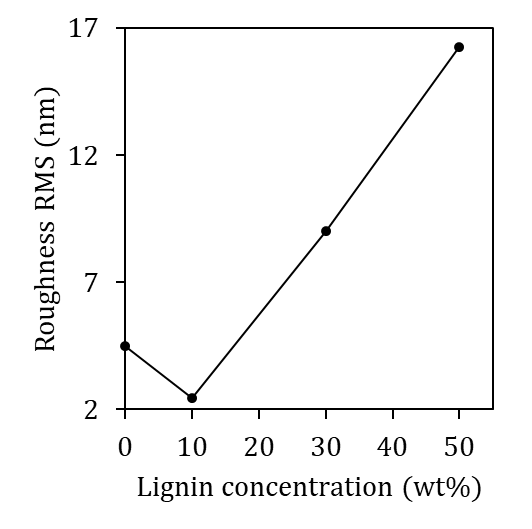


**Figure S19**. Roughness of Lignin films upon lignin concentration.

The plot shows that the roughness of the films increases with the lignin concentration, showing that indeed lignin presence is the leading cause of a spontaneous nanostructured surface after drying.


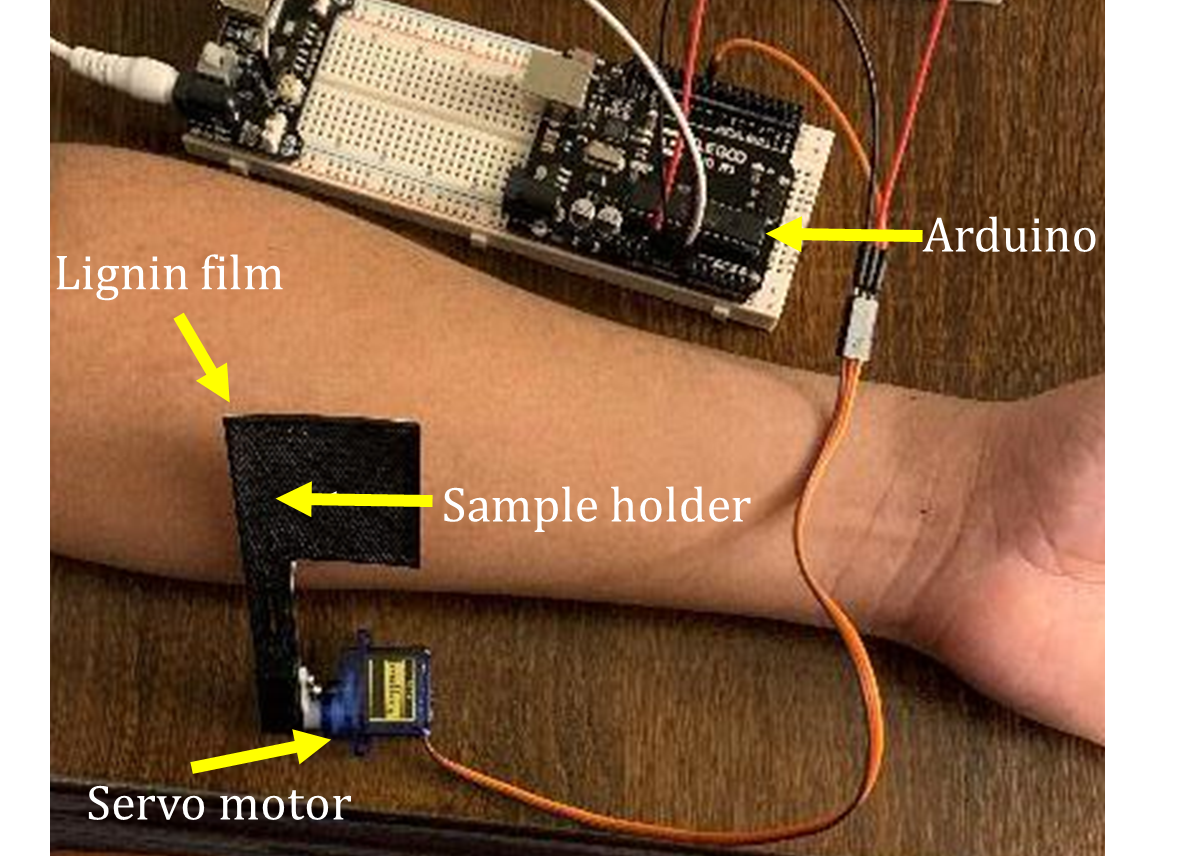


**Figure S20**. **Setup for the characterization of the triboelectric performance of the lignin films against dry skin.** Figure S16 describes the customized setup used to characterize the triboelectric films against dry skin on the forearm (as a counter tribopositive material), where the lignin films were attached to a copper lead for current measurements. Measurements of output voltages in TENGs were conducted with a Keithley 6514 engaged with LabVIEW software. The current outputs were measured by using a low-noise current preamplifier, Stanford Research Systems SR570, following an appropriate configuration. The results of the characteristics are shown in Figure S17. Figure S18 describes the current characteristics of the triboelectric performance of synthetic films against skin as a counter tribopositive material.


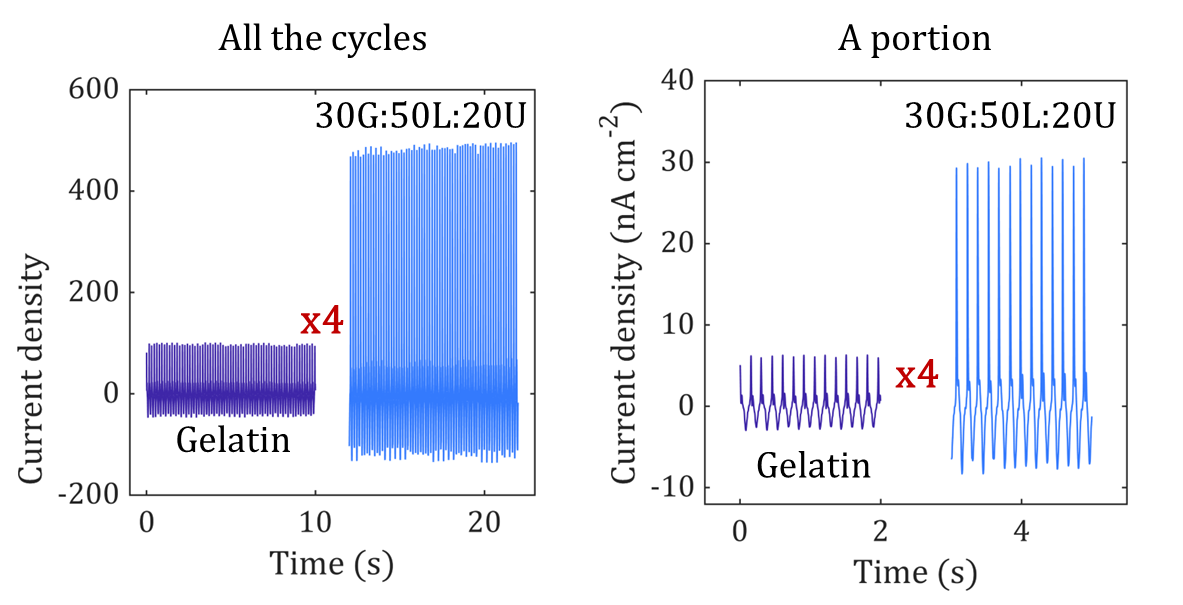


**Figure S21**. **Demonstration of the current output generated by the customized setup to characterize the triboelectric films against dry skin.** The plots show a comparison of the performance of lignin and gelatin-based triboelectric films against dry skin.


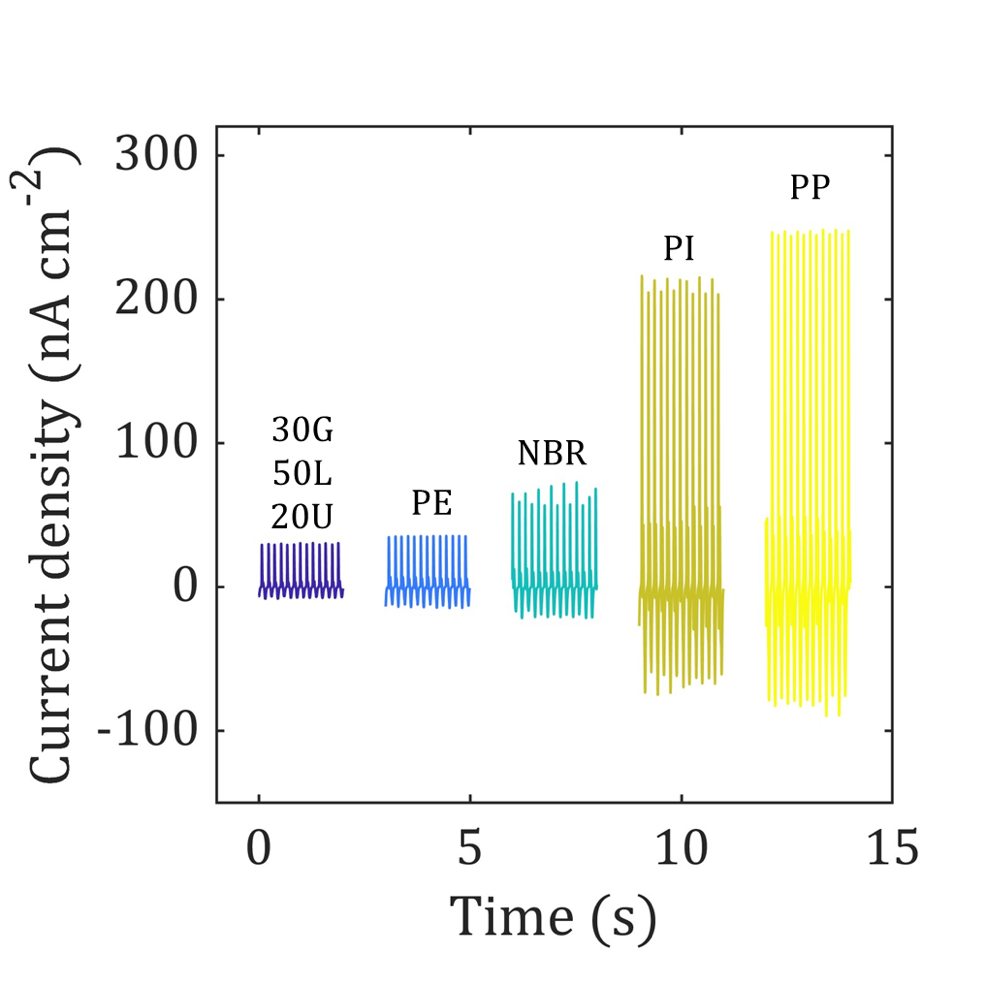


**Figure S22**. Comparison of the performance against dry skin of the 30G:50L:20U sample with polyethylene (PE), acrylonitrile butadiene rubber (NBR), polyimide (PI), and polypropylene (PP).


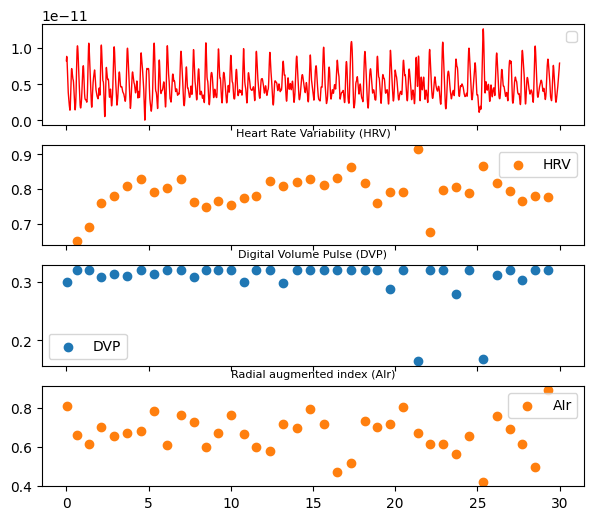


**Figure S23**. **Heart rate variability, differential volumetric pulse, and radial augmented index of R condition**


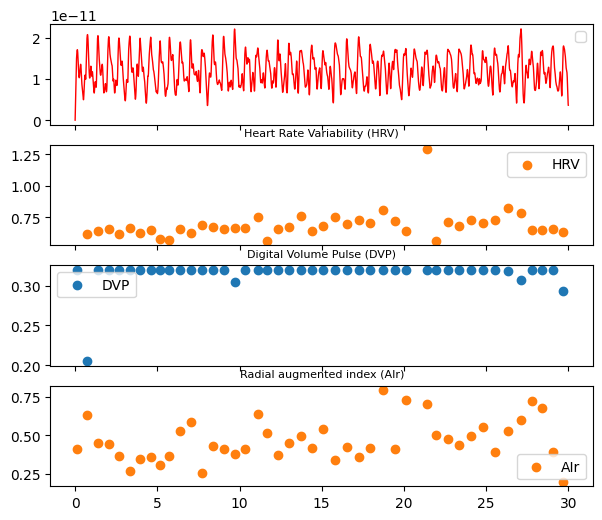


**Figure S24**. **Heart rate variability, differential volumetric pulse, and radial augmented index of WO condition**


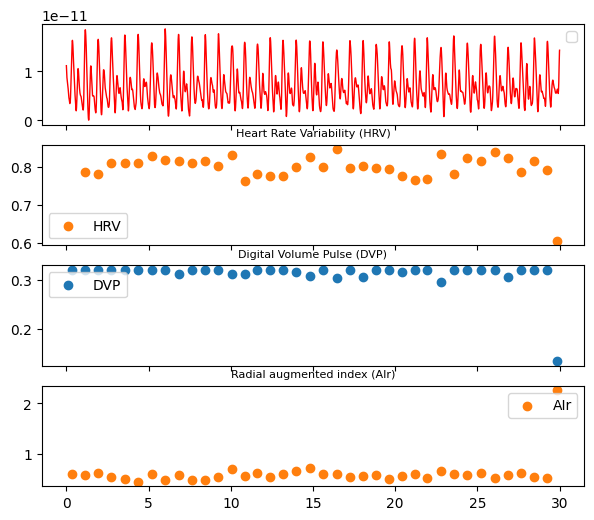


**Figure S25**. **Heart rate variability, differential volumetric pulse, and radial augmented index of S condition**


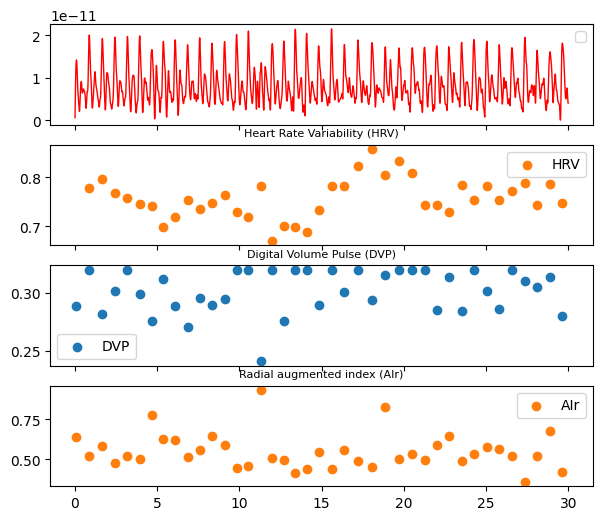


**Figure S26**. **Heart rate variability, differential volumetric pulse, and radial augmented index of F condition**


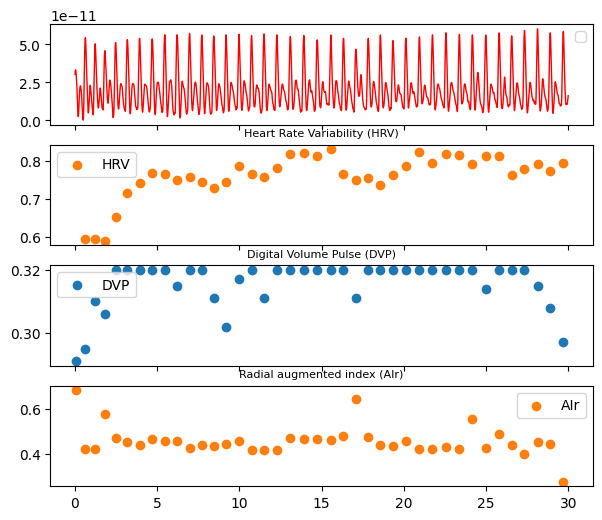


**Figure S27**. **Heart rate variability, differential volumetric pulse, and radial augmented index of EmQ condition**


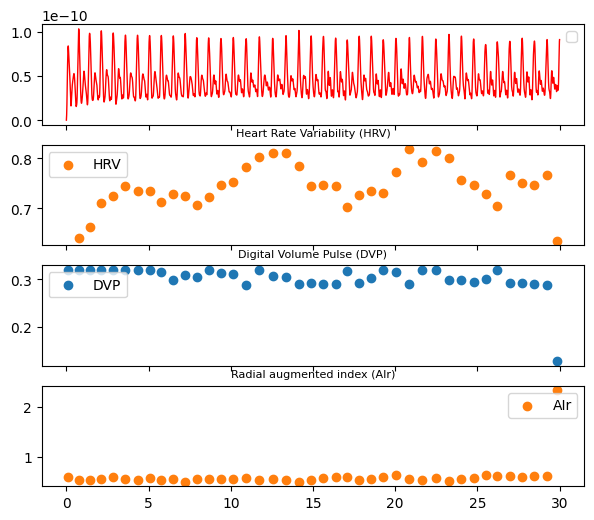


**Figure S28**. **Heart rate variability, differential volumetric pulse, and radial augmented index of InQ condition**


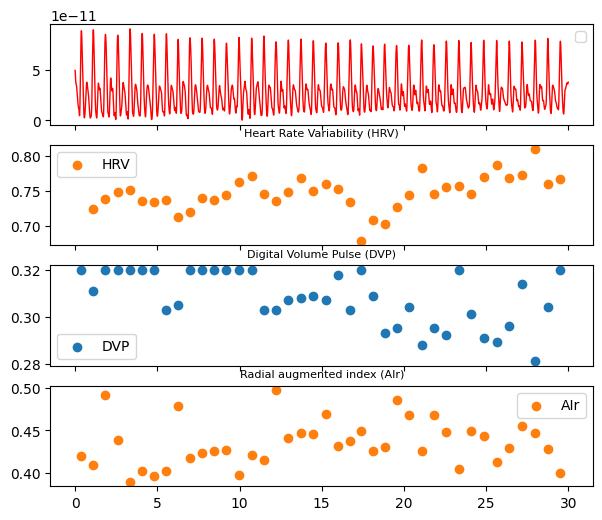


**Figure S29**. **Heart rate variability, differential volumetric pulse, and radial augmented index of MQ condition**


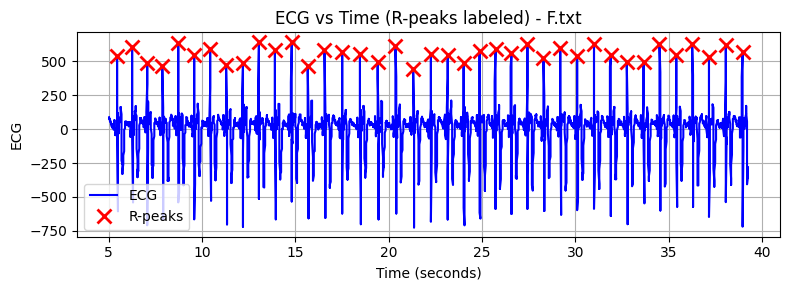


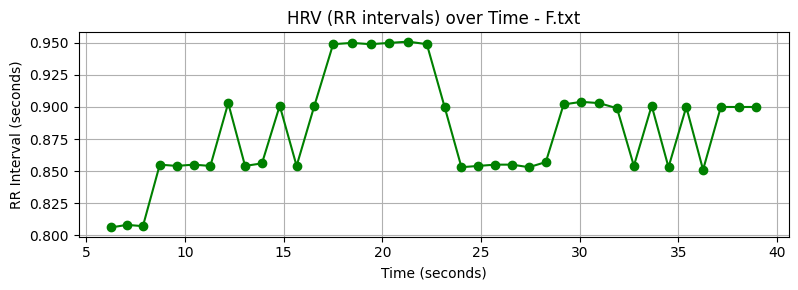


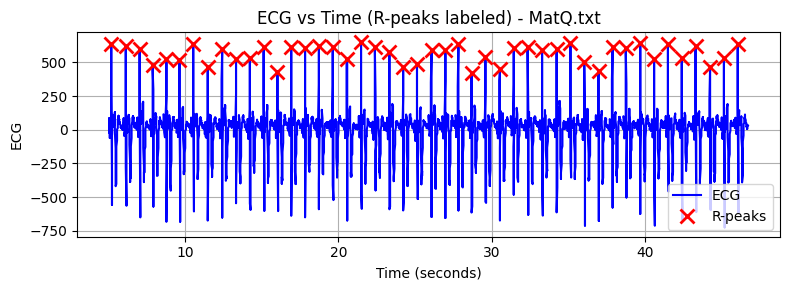


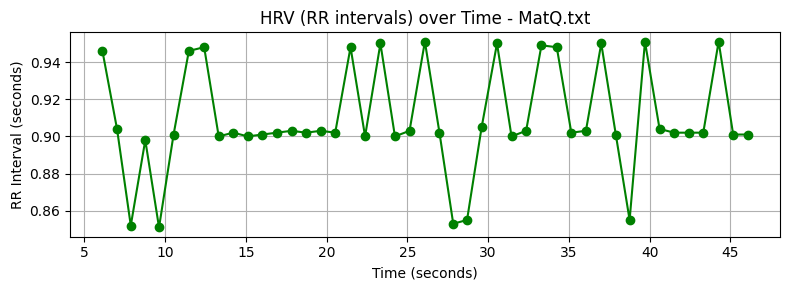


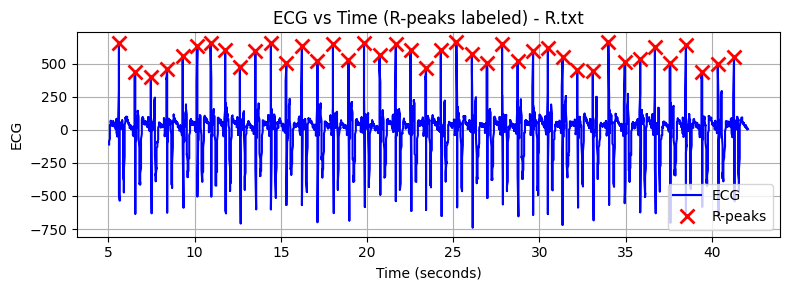


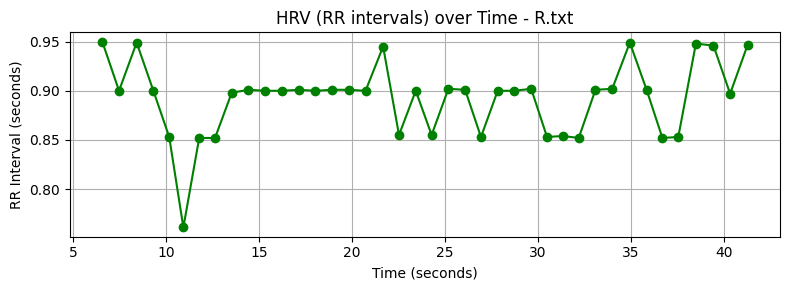


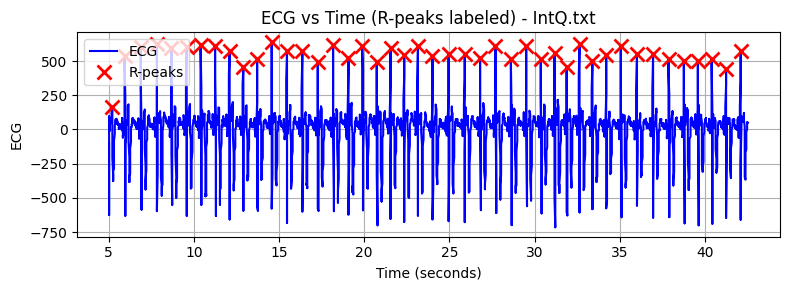


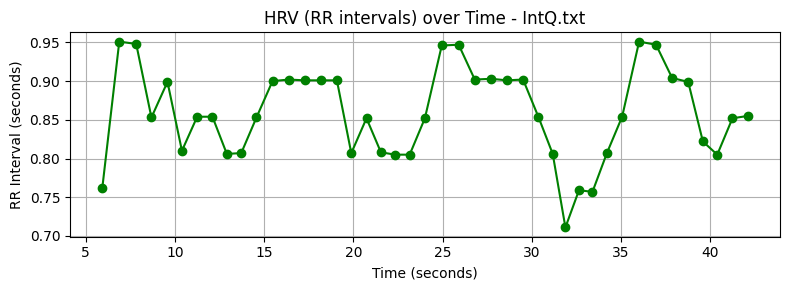


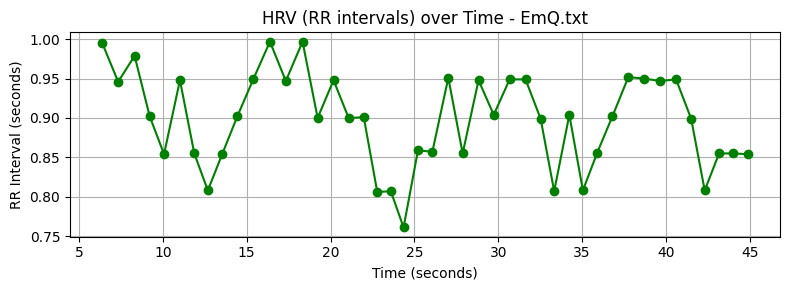


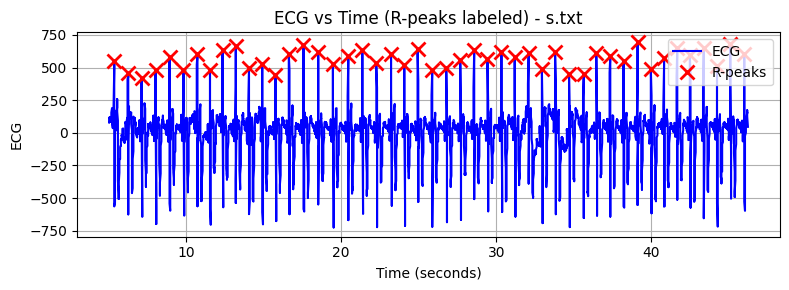


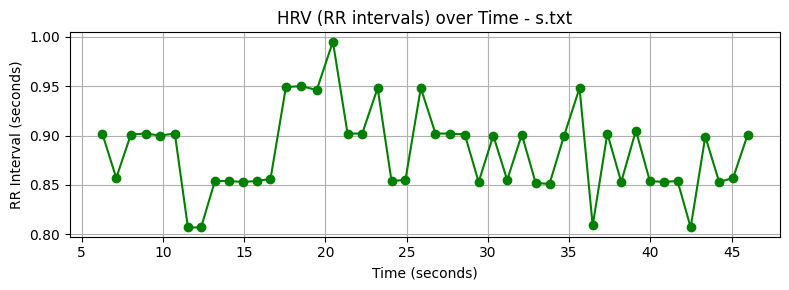


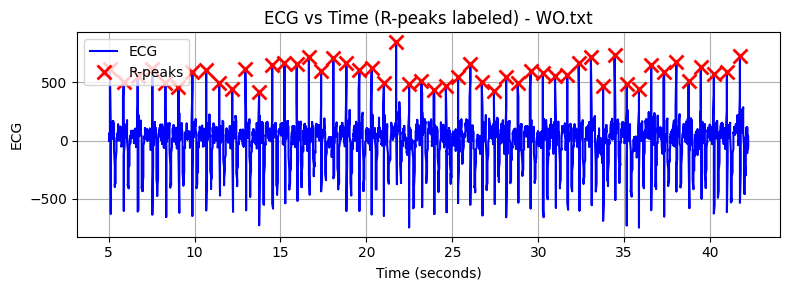


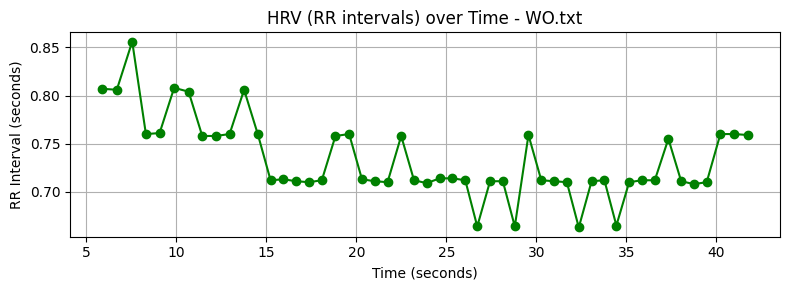


**Figure S30**. **Electrocardiogram (ECG) of the heart rate and heart rate variability under each condition:** Resting (R), working out (WO), singing (S), following (F), emotional questions (EmQ), interview questions (InQ), and mathematical questions (MQ). HRV: Heart rate variability, and SD: standard deviation.


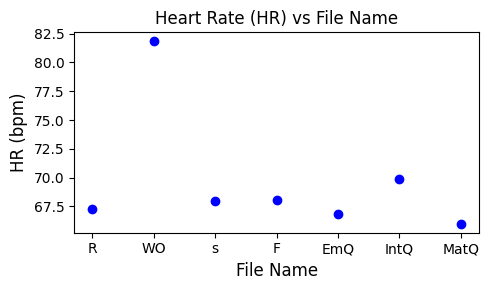


**Figure S31**. **NASA-TLX test measured by the gold standard electrocardiogram (ECG) of the heart rate.**


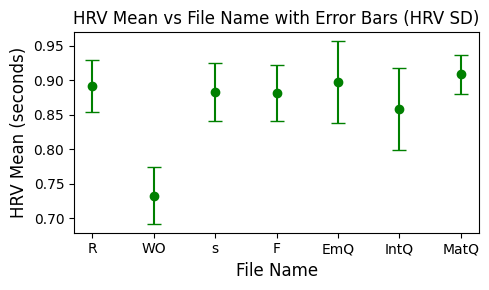


**Figure S32**. **NASA-TLX test measured by the gold standard electrocardiogram (ECG) of the heart rate variability.**


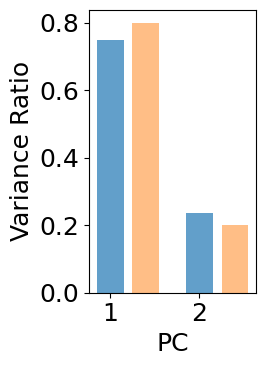


SITS sensor

ECG sensor

0.75

0.24

0.79

0.20

**Figure S33**. **Variance ratio of the SITS and ECG sensors (>95%).** The result demonstrates well dimensionality reduction of the heart rate, heart rate variability and specific cardiovascular hemodynamic metrics of SITS and ECG sensors.

**Supplementary Note 1**

**Hansen Solubility Parameters description**

The Hansen solubility parameters allow formulators to predict which solvents best dissolve certain polymers or which polymers will be compatible with each other, facilitating the design of materials with specific solubility characteristics.

The following equation states that the total solubility parameter squared is the sum of the contributions from dispersive forces, polar forces, and hydrogen bonding. The total solubility parameter is a measure of a substance's ability to dissolve in or mix with another substance, and it comprises these three components.

$$\delta_{T}^{2}=\delta_{D}^{2}+ \delta_{P}^{2}+ \delta_{H}^{2}$$

**Teas graph description**

On a Teas graph, the position of a point relative to the axes reflects the balance of the three types of interactions (dispersive, polar, and hydrogen bonding) that a molecule interacts with. The following three equations define the fractional contributions of each component (dispersive, polar, and hydrogen bonding) to the total solubility parameter.

$$f_{D}= \frac{\delta_{D}}{\delta_{D}+ \delta_{P}+ \delta_{H}}$$

$$f_{P}= \frac{\delta_{P}}{\delta_{D}+ \delta_{P}+ \delta_{H}}$$

$$f_{H}= \frac{\delta_{H}}{\delta_{D}+ \delta_{P}+ \delta_{H}}$$

Each fraction is the respective component's solubility parameter divided by the sum of all three components. These fractions can be useful for understanding the relative importance of each type of intermolecular force in the solubility of a substance.

We can also check whether the fractions of all three components add up to 1 (or 100%) by the following equation:

$$f_{D}+f_{P}+f_{H}=1$$

The Hansen solubility parameter (HSP) δ_t_^2^= δ_D_^2^ + δ_P_^2^ + δ_H_^2^, which indicates the molecular compatibility between solvents and molecules, was used to evaluate the ability of urea to disperse lignin. δ_t_ denotes the total HSP, δ_D_ is the energy of the dispersion forces between molecules, δ_P_ is the energy of the dipolar intermolecular forces between molecules, and δ_H_ is the energy of the hydrogen bonds between molecules. **Figures S1-2** show the representation of the HSP in a Teas graph (the representation of the mathematical fractional contributions of δt and HSP), including the HSP of urea (δ_D_ 22.9, δ_P_ 14.9, and δ_H_ 21.3), lignin (δ_D_ 21.9, δ_P_ 14.1, and δ_H_ 16.9), and other reported solvents for lignin.

**Supplementary Note 2**

**Equations that represent the open-circuit voltage and short-circuit transferred charge of triboelectric sensors.**

The open-circuit voltage of a triboelectric sensor can be given as:

$$V_{OC}(X)=\frac{\sigma x(t)}{\varepsilon_{0}}1$$

The short-circuit transferred charge can be written as:

$$Q_{OC}=\frac{S\sigma x(t)}{d_{0}+x(t)}1$$

**Performance of 1µm films**

Enhanced charge transfer efficiency: A thinner dielectric layer reduces the effective separation distance (d0) between triboelectric surfaces, increasing the interfacial capacitance (C=εA/d0). Under mechanical contact, this allows a larger amount of charge to flow during the contact–separation cycle, resulting in higher short-circuit current. However, excessively thin films (<1 μm) may compromise mechanical integrity or cause dielectric breakdown, so the optimized thickness represents a balance between charge transfer efficiency and film robustness.

Improved charge induction and lower internal resistance: Thinner films facilitate more efficient electrostatic induction because the potential drop across the dielectric is smaller. Consequently, charges generated at the surface can be more effectively transferred to the electrode, minimizing charge trapping or screening within the bulk lignin.

Reduced internal polarization loss: Typically, part of the triboelectric charge is dissipated through dipolar relaxation or trapped within bulk domains, decreasing the effective charge density. Lignin polymer minimizes these losses due to the rigid aromatic molecular structure and enhances charge utilization at the interface.

**Table S1 | Performance benchmarking of wearable triboelectric sensors using biopolymers and synthetic polymers**

| **Reference** | **Tribo-negative material** | **Material Feature** | **Processing Method** | **Tribo-positive material** | **Voc​(V)** | **Isc​**  **(nA cm⁻²)** | **Power Density (µW cm⁻²)** | **Key Application Demonstrated** |
| --- | --- | --- | --- | --- | --- | --- | --- | --- |
| This Work | Lignin-Gelatin | Upcycled Industrial Waste (Kraft Lignin); Biodegradable | Aqueous Blade Coating; Spontaneous Self-Assembly | Skin | >30 | ~30 | 0.02 | Objective Mental Workload Classification (vs. ECG) |
| Wang et al. (2020)  (Ref 1)^1^ | PVA-ionogel | Petrochemical-derived Polymer | Solution Casting | Skin | ~2 | ~20 | ~0.1 | Cardiovascular Monitoring |
| Wang et al. (2018)   (Ref 2)^2^ | Chitosan | Renewable Biopolymer (Chitin) | Laser Processing; Solution Casting | Kapton | ~150 | N.A. | N.A. | Biodegradable Power Source |
| Xiong et al. (2018)  (Ref 3)^3^ | Cellulose Derivative | Renewable Biopolymer (Wood/Cotton) | e.g., Electrospinning, Solution Casting | Skin | ~23 | N.A. | N.A. | e.g., Motion Sensing |
| Zhang et al. (2018)  (Ref 4)^4^ | PTFE | Petrochemical-derived Fluoropolymer; Non-biodegradable**²** | Hot Pressing / Melt Molding | Skin | ~500 | ~160 | 0.4 | Biomechanical Energy Harvesting |
| Jayaweera et al. (2018) (Ref 5)^5^ | Kapton | Petrochemical-derived Polymer | Spin coating | Human Hair | 103 | N.A. | N.A. | Biomechanical Energy harvester |
| Yang et al. (2013) (Ref 6)^6^ | PDMS | Petrochemical-derived Polymer | Etching and laser cutting | Skin | N.A. | ~800 | N.A. | Tactile sensor |

*¹ Total current reported, not normalized by area. Direct comparison of current density is not possible.*

*² Polytetrafluoroethylene (PTFE) is a fluoropolymer with concerns regarding environmental persistence and potential biocompatibility issues related to per- and polyfluoroalkyl substances (PFAS).*

**Table S2**. Detailed NASA-TLX survey per condition: Resting (R), working out (WO), singing (S), following (F), emotional questions (EmQ), interview questions (InQ), and mathematical questions (MQ).

| Condition | Survey |
| --- | --- |
| Resting | The measurement is taken during a person sitting in a chair. |
| Working out | Lifting with one hand a 10 Kg weight while seated in a chair. |
| Singing | The participant is asked to sing any song that they might like while seated in a chair. |
| Following | The participant is asked to follow the path of a snake in a recorded snake game, while seated in a chair. |
| Emotional questions | The following questions are asked while the participant is seated in a chair, giving 20 seconds to answer each question:  Do you have dreams?  Do you have any regrets?  Do you regret meeting someone in your life? |
| Interview questions | The following questions are asked while the participant is seated in a chair, giving 20 seconds to answer each question:  How much do want to earn?  What are your virtues at work? Explain why?  Can you list your defects at work? Explain why? |
| Mathematical questions | The following questions are asked while the participant is seated in a chair, giving 5 seconds to answer each question:  11/2  2+2  4+1420  7+11  36+78  4*7  12*6  25*7  16/5  4^4 |

We used the six aspects recommended by the subjective NASA-TLX test, with each aspect ranked from 1 to 6 as recommended. The six aspects considered were mental demand (MD), physical demand (PD), temporal demand (TD), performance (P), effort (E), and frustration (F). In the case of MW conditions, we measured cardiovascular activity during resting (R), working out (WO), singing (S), following (F), emotional questions (EmQ), interview questions (InQ), and mathematical questions (MQ).

**Table S3**. Statistics of the heart rate measured under each condition: Resting (R), working out (WO), singing (S), following (F), emotional questions (EmQ), interview questions (InQ), and mathematical questions (MQ). HRV: Heart rate variability. DVP: differential volumetric pulse. AIr: Radial augmented index. SD: standard deviation.

|  | R | WO | S | F | EmQ | InQ | MQ |
| --- | --- | --- | --- | --- | --- | --- | --- |
| BPM | 76 | 90 | 76 | 80 | 80 | 82 | 80 |
| HRV Mean | 0.79 | 0.67 | 0.80 | 0.76 | 0.76 | 0.74 | 0.75 |
| HRV SD | 0.05 | 0.07 | 0.04 | 0.04 | 0.06 | 0.04 | 0.02 |
| DVP Mean | 0.31 | 0.17 | 0.31 | 0.30 | 0.32 | 0.30 | 0.31 |
| DVP SD | 0.03 | 0.01 | 0.03 | 0.02 | 0.01 | 0.03 | 0.01 |
| AIr Mean | 0.67 | 0.68 | 0.61 | 0.55 | 0.46 | 0.61 | 0.44 |
| AIr SD | 0.10 | 0.14 | 0.28 | 0.11 | 0.06 | 0.27 | 0.03 |

**Table S4**. Statistics of the ECG heart rate and heart rate variability measured under each condition: Resting (R), working out (WO), singing (S), following (F), emotional questions (EmQ), interview questions (InQ), and mathematical questions (MQ). HRV: Heart rate variability, and SD: standard deviation.

| MW condition | HR (bpm) | HRV Mean (seconds) | HRV SD (seconds) |
| --- | --- | --- | --- |
| F | 68.0597 | 0.881579 | 0.040233 |
| MatQ | 66.00983 | 0.908956 | 0.028421 |
| R | 67.25138 | 0.892175 | 0.037775 |
| IntQ | 69.89029 | 0.858488 | 0.05911 |
| EmQ | 66.88116 | 0.897114 | 0.059425 |
| s | 67.96356 | 0.882826 | 0.042171 |
| WO | 81.82636 | 0.73326 | 0.040682 |

**References**

1. Wang, R. *et al.* Holistically Engineered Polymer–Polymer and Polymer–Ion Interactions in Biocompatible Polyvinyl Alcohol Blends for High-Performance Triboelectric Devices in Self-Powered Wearable Cardiovascular Monitorings. *Adv. Mater.* **32**, 1–10 (2020).

2. Wang, R. *et al.* Engineered and Laser-Processed Chitosan Biopolymers for Sustainable and Biodegradable Triboelectric Power Generation. **1706267**, 1–8 (2018).

3. Xiong, J. *et al.* Skin-touch-actuated textile-based triboelectric nanogenerator with black phosphorus for durable biomechanical energy harvesting. *Nat. Commun.* **9**, 4280 (2018).

4. Zhang, R. *et al.* Harvesting triboelectricity from the human body using non-electrode triboelectric nanogenerators. *Nano Energy* **45**, 298–303 (2018).

5. Jayaweera, E. N., Wijewardhana, K. R., Ekanayaka, T. K., Shahzad, A. & Song, J.-K. Triboelectric Nanogenerator Based on Human Hair. *ACS Sustain. Chem. Eng.* **6**, 6321–6327 (2018).

6. Yang, Y. *et al.* Human Skin Based Triboelectric Nanogenerators for Harvesting Biomechanical Energy and as Self-Powered Active Tactile Sensor System. *ACS Nano* **7**, 9213–9222 (2013).
